# Validation framework for epidemiological models with application to COVID-19 models

**DOI:** 10.1371/journal.pcbi.1010968

**Published:** 2023-03-29

**Authors:** Kimberly A. Dautel, Ephraim Agyingi, Pras Pathmanathan

**Affiliations:** 1 School of Mathematical Sciences, Rochester Institute of Technology, Rochester, New York, United States of America; 2 Division of Biomedical Physics, Office of Science and Engineering Laboratories, Center for Devices and Radiological Health, Food and Drug Administration, Silver Spring, Maryland, United States of America; Yale School of Public Health, UNITED STATES

## Abstract

Mathematical models have been an important tool during the COVID-19 pandemic, for example to predict demand of critical resources such as medical devices, personal protective equipment and diagnostic tests. Many COVID-19 models have been developed. However, there is relatively little information available regarding reliability of model predictions. Here we present a general model validation framework for epidemiological models focused around predictive capability for questions relevant to decision-making end-users. COVID-19 models are typically comprised of multiple releases, and provide predictions for multiple localities, and these characteristics are systematically accounted for in the framework, which is based around a set of validation scores or metrics that quantify model accuracy of specific quantities of interest including: date of peak, magnitude of peak, rate of recovery, and monthly cumulative counts. We applied the framework to retrospectively assess accuracy of death predictions for four COVID-19 models, and accuracy of hospitalization predictions for one COVID-19 model (models for which sufficient data was publicly available). When predicting date of peak deaths, the most accurate model had errors of approximately 15 days or less, for releases 3-6 weeks in advance of the peak. Death peak magnitude relative errors were generally in the 50% range 3-6 weeks before peak. Hospitalization predictions were less accurate than death predictions. All models were highly variable in predictive accuracy across regions. Overall, our framework provides a wealth of information on the predictive accuracy of epidemiological models and could be used in future epidemics to evaluate new models or support existing modeling methodologies, and thereby aid in informed model-based public health decision making. The code for the validation framework is available at https://doi.org/10.5281/zenodo.7102854.

## Author summary

During the COVID-19 pandemic many mathematical models have been developed. These mathematical models provide forecasts of key quantities such as number of infectious cases, number of hospitalizations, and number of deaths, in the upcoming weeks in the locality of interest. However, the reliability of model predictions is unclear. Currently, there have been few techniques employed to validate the performance of COVID-19 models that have focused on quantities that are especially of interest to end-users, such as when deaths will peak or the magnitude of the peak. Here, we provide an epidemiological model validation framework focused on questions relevant to decision-makers that utilize COVID-19 model predictions. We analyze four COVID-19 models with our framework, examining the accuracy of each model in predicting the date and magnitude of a peak, recovery rate, and monthly cumulative counts, for predictions of deaths and of hospitalizations. Our results show that the mathematical models produce highly variable predictions across regions. Our framework demonstrates the need for predictive reliability of epidemiological models.

## Introduction

### Background

Mathematical models provide insight during public health emergencies. This was especially true when the coronavirus disease (COVID-19) outbreak became pandemic and public health officials looked towards mathematical models as a key source of information. Mathematical models offer predictions on the near- and medium-term evolution of the pandemic, allowing researchers and policymakers to plan responses at the local and national levels [1, 2]. Models are powerful tools as they can predict infectious cases, deaths, and hospitalizations [3–7]. From these, information can be inferred regarding demand of vital resources such as personal protective equipment (PPE), ventilators, and diagnostic tests [8–10]. Additionally, critical decisions concerning lockdowns and social distancing policy can be informed by modeling predictions [11, 12].

At the onset of the pandemic several models emerged exploring the transmission dynamics of COVID-19 [3], and many more models were developed over the course of the pandemic. Different models focused on different localities, whether one region, multiple US states [4, 5], or potentially multiple countries [6, 7]. Modeling groups released updated predictions on various timelines, varying from daily new predictions, every few days, to weekly new predictions. Various modeling approaches were utilized, including compartmental models [11, 13], statistical models [6, 14], agent-based models [15, 16], and machine learning models [5, 17]. Since April 2020 the U.S. Centers for Disease Control and Prevention (CDC) has partnered with various research groups to advance the understanding of COVID-19. Collecting results from the modeling groups every week, the CDC released predictions on their website of the expected weekly number of new cases, deaths and hospitalizations, for the next four weeks. This allowed for one collective website where numerous models and their predictions are available and can be compared. In June 2020 the CDC then created and shared an ensemble model which combines each of the independently-developed predictions into one aggregate prediction over the next four weeks [2].

The duration of forecasts varied across models and release date. Early models provided longer duration (e.g. > 6 weeks) predictions [5, 6]. Later on, many models and the CDC chose only to provide short-term (e.g. 4 weeks) predictions [2]. While short-time horizon models can be useful, models that are reliably able to forecast on longer horizons are more helpful for long-term public health decision making. Health authorities can identify the features of the virus’s spread and create effective prevention and containment plans in advance by forecasting the epidemic’s long-term trajectory [18]. Long-term forecasts are difficult, however, since the status of the pandemic is regulated by human action and interaction. Rahmandad et al. [19] demonstrates that an endogenous depiction of human behavior in interaction with the growing pandemic is essential for epidemic models to have long-term prediction value.

With so many emerging models it is difficult to know which of their forecasts are reliable. There are many challenges to modeling COVID-19 progression, leading to considerable uncertainty in model reliability. Depending on the modeling approach and what measures are included, models may capture different dynamics and have varying predictive capabilities. There are two main sources of uncertainty within each model: uncertainty in the disease evolution and consequently on the appropriateness on the equations used, and uncertainty in the model parameters. Depending on the approach to estimate parameters, a range of values will be obtained for parameters such as incubation rate or recovery rate. Furthermore, each specific outbreak is uniquely occurring at a specific time and location, which is subject to various local factors and conditions—environmental, population, social, and others.

One important factor impacting reliability of epidemiological models is model transparency. It is important that model results are not presented in isolation and model code is made publicly available so the results can be replicated and evaluated [20]. A lack of transparency among COVID-19 models can lead to misunderstanding, misuse, or deliberate misinformation about models and their results. Scientists are prevented from confirming the results and enhancing the model’s functionality because there is a lack of transparency in the creation, development, and analysis of these models [21]. This diminishes the trust in the model’s timely message and limits the model’s use. To address the need for model transparency during the COVID-19 pandemic Jalali et al. [21] assessed 29 COVID-19 models. Transparency assessment criteria included specificity of model items, such as discussion of assumptions, parameterization, codes, and sensitivity analyses, along with general research items, such as disclosure of research limitations, funding, and potential conflicts of interest. Jalali et al. [22] further comments that journals can and should continue to contribute to the prioritization of transparency among models.

Another important factor impacting reliability of epidemiological models is model credibility—that is, trust, based on all sources of evidence, in the predictive capability of the model [23]. If mathematical models are used in public health decision-making, it is critical that decision-makers are provided information on model credibility. Model credibility is primarily achieved by performing model validation, which is comparison of model outputs against real-world data not used in the development of the model. For COVID-19 models, this involves comparing predicted local cases, deaths and/or hospitalizations against reported values of these quantities. However, there are numerous challenges faced when validating COVID-19 models. One is that there is no single set of predictions but rather multiple releases of each model, each a new set of predictions. Further, most models provide predictions for multiple regions. Certain models allow for predictions on the county level, state level, and national level. Validating a single model release for a single region, against observations, is conceptually straightforward. However, defining a rational and comprehensive validation strategy for validating all model releases and all regions of interest is not. The ‘true’ data also presents several challenges. There is uncertainty in the daily counts as reporting processes vary among locations, and there are daily fluctuations and other noise in the data. For deaths and hospitalizations, reported numbers are impacted by how often organizations choose to release data. For example, some organizations may not report weekend deaths and add them to the count for the following Monday. For COVID-19 cases, there is uncertainty in undiagnosed cases, or later unreported cases with at-home test kits. These points cause challenges when comparing COVID-19 models to data. Challenges also arise pertaining to comparison between different models. Different groups provide new releases on different schedules, making a fair comparison difficult.

The Center for Devices and Radiological Health (CDRH) at the U.S. Food and Drug Administration (FDA) is responsible for ensuring safety and effectiveness of medical devices marketed in the U.S. CDRH is developing capabilities to strengthen public health supply chains by proactively monitoring and assessing risks and vulnerabilities to prevent shortages of medical devices. Predictive models of acute demand of medical devices (e.g., ventilators, PPE or diagnostic tools) during public health emergencies are expected to play an increasing role in preventing shortages. Demand models are closely related to epidemiological models, with outputs from the latter often informing the former. For example, the WHO COVID-19 Essential Supplies Forecasting Tool [8] provides the user with a choice among several epidemiological methods for forecasting COVID-19 cases, including an integration with Imperial College’s Susceptible-Exposed-Infectious-Removed (SEIR) model [12]. Wells et al. [9] projected the demand for ventilators at the peak of the COVID-19 outbreak in the USA by combining an age-structured dynamic model of SARS–CoV-2 transmission and current data [24]. McCabe et al. [10] integrates hospital capacity planning and epidemiological projections of COVID-19 patients to estimate the demand for and resultant spare capacity of intensive care unit beds, staff and ventilators. Therefore, assessing accuracy of the underlying epidemiological model (whether COVID-19 or a future epidemic) is critical to understanding accuracy of demand models.

### Previous work validating COVID-19 models

Various techniques have been employed to validate the performance of COVID-19 models. Such methods include comparing model predictions against observed values using mean absolute error (MAE) or mean absolute percentage error (MAPE), weighted interval score (WIS) and others.

Ray et al. [25] evaluated the performance for the CDC ensemble model, weekly for each state using the MAE as a measure of total error, as well as analyzing the number of observations falling within predictive intervals. Similarly, Konarasinghe in [26], validation was performed for a model of the COVID-19 epidemic in India and Brazil using MAPE, mean square error, and mean absolute deviation as validation metrics. Atchadé and Sokadjo [27] analyzed three univariate models which used daily world infectious data and performed cross validation among the three models as well as calculating MAPE for each model. Ramazi et al. [28] forecasted COVID-19 mortality in the US and also used MAPE to evaluate predictive accuracy as well as compare their model with others shared with the CDC. An agent-based model simulating the spread of COVID-19 in a city was developed in Shamil et al. [29] and validated by comparing the simulation to the real data of Ford County, Kansas, using root mean squared error. None of these cases involved simultaneous validation of multiple releases of a model. In contrast, Jin et al. [30] performed validation of the IHME model for New York and Italy, comparing the accuracy of three different model releases in predicting date of peak deaths. The only analysis we are aware of that accounts for different model releases in a systematic way is Friedman et al. [31], where members of the COVID-19 Forecasting Team within the Institute for Health Metrics and Evaluation introduced a publicly available evaluation framework for assessing the predictive validity of COVID-19 mortality forecasts. Seven models that were global in scope, and provided public date-versioned forecasts, were analyzed. Median absolute percent error values, a measure of accuracy, were calculated across all observed errors at weekly intervals, for each model by week of forecasting and geographic region to analyze peak magnitude predictions. Additionally, each model was assessed on how well they predicted the timing of peak daily deaths.

Many forecasts are issued in the form of central predictive intervals at various levels. The WIS is a well-known quantile-based proper score that approximates the continuous ranked probability score [32]. It can be interpreted as a generalization of the absolute error to probabilistic forecasts and allows for a decomposition into a measure of sharpness and penalties for over- and under-prediction. Carnegie Mellon University Delphi Group validates each model shared with the CDC as well as their ensemble model, for weekly state forecasts using the weighted interval score. These model validation results are computed weekly and provided to the public [33]. This website complements a related website which allows users to compare model predictions against reported values, visually and interactively, for the CDC models [2]. However, because of the limited time horizon used in the CDC initiative (model predictions for the next four weeks only), these efforts only provide information on short-term model accuracy.

Rahmandad et al. [19] concentrated their studies on the relationship between a model’s structure and its predictive accuracy, using the findings to create their own model and evaluate its predictive performance. Analyzing 61 models and weekly death predictions from the CDC forecast hub, each model was classified as one of four categories: mechanistic compartmental, non-mechanistic, ensemble, and other. Predictive accuracy was measured using the absolute prediction error normalized by a location’s population, comparing each model type with a constant model using linear regression with location-forecast date-forecast horizon fixed effects. Rahmandad et al. [19] found differences in performance in the short-term and long-term based upon the type of model. Non-mechanistic and ensemble models outperformed compartmental models that do not benefit from state-resetting in the short-term, but the rankings changed after 4–5 weeks of projection horizon [19]. On average, compartmental models with state-resetting outperformed in both the short- and long-term [19].

Many have commented on the difficulty of validating models and the lack of validation performed and provided recommendations on how to use the models best for information. Eker [1] examined three models—the Imperial College London model, the IHME model and the Austrian COVID-19 model—and commented on the little validation shared on these models, even though all three were used to make public health policy decisions. The analysis provided in Jin et al. [30], mentioned above, was performed as part of an argument for greater model transparency, reproducibility, and validity assessment. Finally, Islam et al. [34] argues that there is a “crucial need to develop a framework that includes transparency, reproducibility, and a prospective validation to evaluate COVID-19 projection models.”

Although the focus of this work is on validating COVID-19 models, it is important we pause and remark here that attempts at providing a general framework for validating epidemiological models are still at the infancy level. The recent work of Jalali et al. [35] addresses the currents gaps towards a broader framework by identifying and analyzing a wide variety of published research models in health policy and epidemiology. The analysis in Jalali et al. [35] includes a broad comparison of model techniques, a systematic assessment that focuses on design, implementation, validation, dissemination and the reproducibility of a model.

### Aims

The aim of this paper is to present a general model validation framework for multi-release multi-location epidemiological models, focused on questions relevant to decision-making end-users, and further to apply this framework to evaluate the accuracy of COVID-19 models. As discussed above, we are not aware of any validation framework that accounts for multiple model releases and localities in a systematic way, other than Freidman et al. [31] which considers releases systematically and Carnegie Mellon University Delphi Group [33] which validates the limited weekly four-week ahead predictions provided to the CDC. (Differences between our work and Friedman et al. [31] will be discussed in the Discussion section). Moreover, metrics such as MAE, MAPE and WIS, while important, do not provide direct information to users on the accuracy of models’ ability to predict key quantities of interest. Key quantities of interest that are relevant to public health decision makers include expected date that cases/deaths/hospitalization will peak, expected magnitude at the peak, expected total number of cases/deaths/hospitalizations over a fixed time period, and expected time to recovery.

Therefore, in this paper we present a novel framework for assessing the reliability of COVID-19 models. The framework is also applicable to other epidemiological models and related models such as resource demand models: it is applicable to any model that provides regularly updated predictions of the value of some quantity (e.g., deaths/hospitalization) over a medium-term (e.g., > 6 weeks) time horizon. The framework is based around a set of validation metrics that quantify model accuracy of quantities of interest such as date of peak, magnitude of peak, and recovery. The framework systematically deals with multiple model releases in a manner that allows comparison between models with releases on different schedules, and systematically handles multiple regions. We apply our framework to retrospectively evaluate the predictive performance of four major COVID-19 models. However, our framework and the supporting tool will be most useful in the development of new models in future public health emergencies.

## Methods

### Overview

Our approach can be applied to any model that predicts the future daily value of some quantity across multiple localities, where the quantity goes through waves of increased value, and the full set of model predictions is made up of multiple releases generated on different dates. We apply it on COVID-19 models of expected daily deaths and hospitalizations, across U.S. states. We have chosen not to analyze accuracy of predicted COVID-19 cases because of the large uncertainty in true case counts, especially at the beginning of the pandemic.

**Fig 1:**
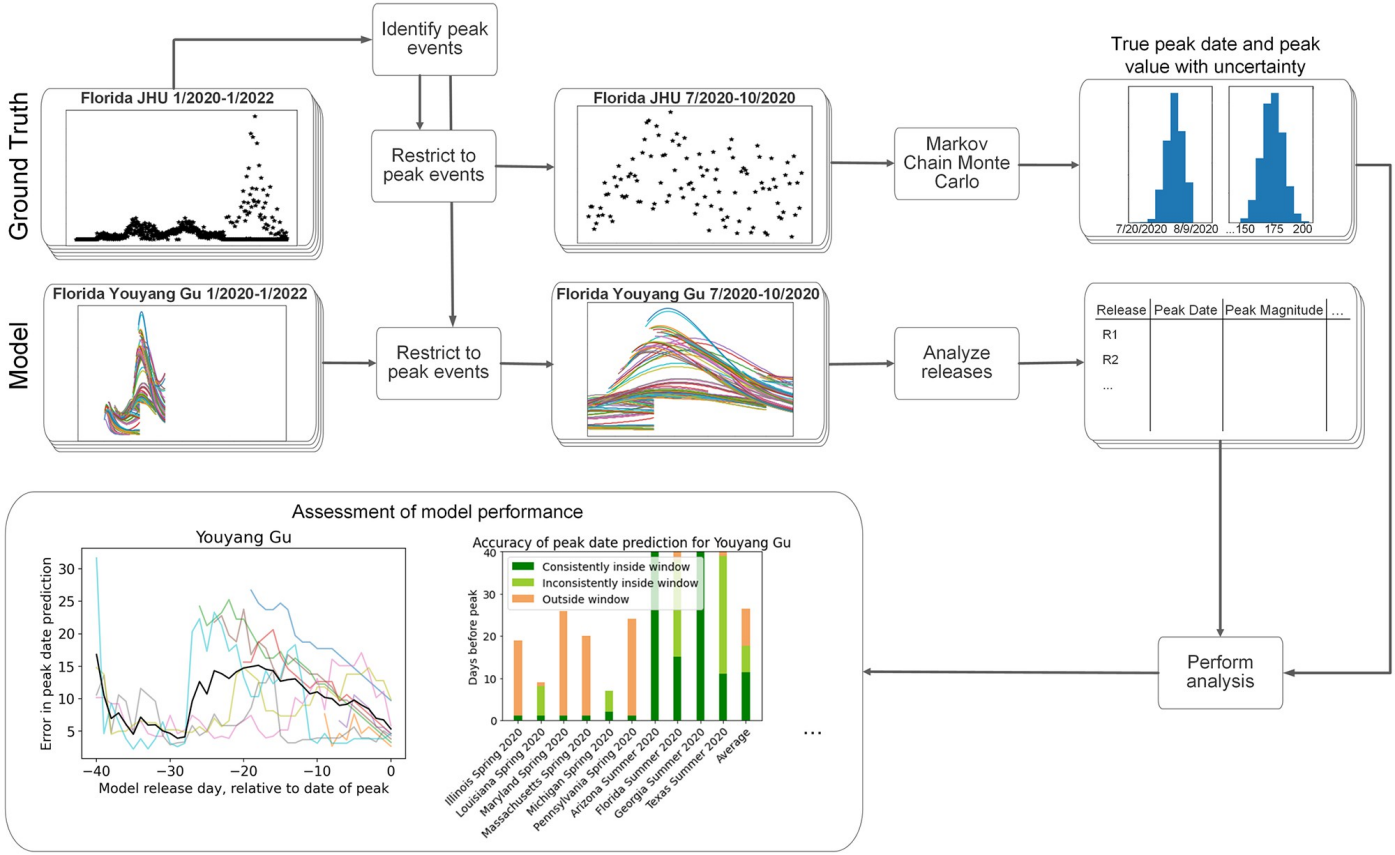
Workflow of model validation framework. Peak events are identified from the ground truth, which are then analyzed to determine peak dates and peak magnitudes (and other quantities). Uncertainty in this quantities is accounted for. Simultaneously, model predictions for the peak events are analyzed, and each release’s prediction of peak date and magnitude is identified. The collected information are then analyzed to obtain measures of the performance of the model for each peak event.

Fig 1 provides an overview of our approach. The starting points are the ground truth dataset and the model predictions. The ground truth is composed of the observed daily deaths/hospitalizations for each US state. The model predictions are composed of a set of releases, with each release providing predictions for some time window for each US state. We first identify a set of ‘peak events’ (i.e., local COVID-19 ‘waves’) satisfying certain pre-defined criteria. The ground truth data is restricted to each peak event and analyzed to identify characteristics of the peak event (quantities of interest—QOIs). Example QOIs include date of peak, magnitude of peak, and time to recovery. Since there is significant uncertainty in the true values of these QOIs from the ground truth dataset, due to underlying stochasticity and reporting delays, we fit a statistical model using the Markov Chain Monte Carlo (MCMC) method to obtain posterior distributions for the true QOIs. The corresponding model predictions of each QOI—for each release—are then identified. We define validation metrics that assess how well the totality of the model predictions predicted the QOIs. The result is a set of values for each peak event, that characterizes how well the model in total predicted that peak event and can be directly interpreted by the end user. We can then average across the peak events to obtain measures that summarize the overall performance of the model in the US.

### Notation and dummy releases

Let us introduce some terminology and notation. A model is all predictions released by a given organization. A model is comprised of multiple model releases, *m*_*i*_, which is a specific prediction released on date *d*_*i*_, made up of predicted daily deaths/hospitalizations for regions of interest (in our case, U.S. states) from day *d*_*i*_ + 1 until an end date that varies between models and releases (typical prediction windows range from a few weeks to a few months). Since modeling organizations release predictions using different schedules, we assign dummy intermediate releases using the most recent release. That is, if the organization releases predictions $m_{d_{1}}$ on day *d*_1_, $m_{d_{2}}$ on day *d*_2_, etc., we assume daily releases of the form $\left(m_{d_{1}},m_{d_{1}},\ldots ,m_{d_{1}},m_{d_{2}},m_{d_{2}},\ldots m_{d_{2}},m_{d_{3}},\ldots \right)$. Some of the validation scores defined below require a model release to be defined for every day within a fixed range of dates, which is why dummy releases need to be defined.

### Data sources and models

Our ground truth data source of daily deaths due to COVID-19 was the COVID-19 Data Repository by the Center for Systems Science and Engineering at Johns Hopkins University [36]. For hospitalization data, we used the COVID Tracking Project [37].

The models we analyzed were based on which groups provided sufficient information for us to apply our framework. Our framework requires access to the raw model predictions for the latest release and all previous releases. Unfortunately, few modeling groups make the previous predictions publicly available. We went through all 33 models listed on the CDC website as of July 28, 2021 [38] and identified four models that satisfied these criteria for daily death predictions for US states: (i) Institute for Health Metrics and Evaluation (IHME) model [7]; (ii) Los Alamos National Laboratory (LANL) model [6]; (iii) U. Texas Austin model [4]; (iv) Youyang Gu (YYG) model [5]. Just one model that provided US state hospitalization predictions satisfied these criteria, the IHME model. Table 1 provides some information on these models.

**Table 1:** List of models evaluated.

Name	Modeling approach	Model output	First release	Last release
Institute for Health Metrics and Evaluation (IHME) [7]	Co-variate driven SEIR combined with spline	Cases, hospitalizations, and deaths	March 25^*th*^, 2020	Ongoing (as of May 4^*th*^, 2022)
Los Alamos National Laboratory (LANL) [6]	Statistical dynamical growth	Cases and deaths	April 5^*th*^, 2020	September 27^*th*^, 2021
U. Texas Austin (UTexas) [4]	Bayesian multilevel negative binomial regression model	Deaths	April 14^*th*^, 2020	April 26^*th*^, 2021
Youyang Gu (YYG) [5]	SEIR combined with machine learning	Cases and deaths	April 2^*nd*^, 2020	October 4^*th*^, 2020 (deaths)

### Selection of peaks

**Fig 2:**
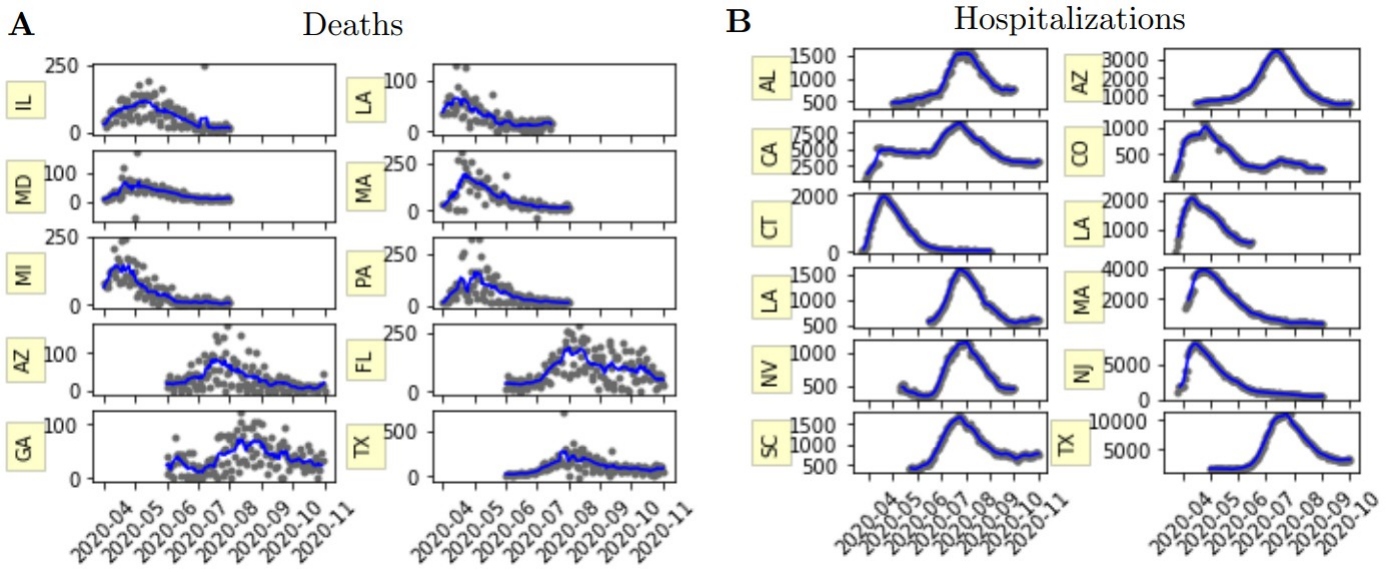
A) Johns Hopkins University and B) COVID Tracking Project data during spring and summer 2020 for respective death and hospitalization peak events. Gray dots represent daily data and blue line represents 7 day rolling average.

We selected a set of peak-events for validating the models as follows. Daily death/hospitalization time series for each state were assessed for candidate events by eye, for periods with a clear rise and decrease in deaths/hospitalizations. Candidate events were retained as peak events if they satisfied the following: (i) seven-day average maximum was greater than 50 (deaths) or 1000 (hospitalizations); (ii) seven-day average was less than 50% of maximum value in period prior to peak; (iii) seven-day average decreased to less than 50% of maximum value in period after peak. We considered 2020 spring and summer events only since there were limited model predictions available for the following fall and winter peak events (Table 1). Table 2 lists the identified peak events, and they are plotted in Fig 2.

**Table 2:** Peak events for daily deaths and hospitalizations.

Output	State	Period	Comments
Daily deaths	Illinois	Spring 2020	
	Louisiana	Spring 2020	
	Maryland	Spring 2020	
	Massachusetts	Spring 2020	
	Michigan	Spring 2020	
	New York	Spring 2020	Occurred before most models had been developed so not included in validation
	Pennsylvania	Spring 2020	
	Arizona	Summer 2020	
	Florida	Summer 2020	Does not recover sufficiently to compute recovery metrics
	Georgia	Summer 2020	Does not recover sufficiently to compute recovery metrics
	Texas	Summer 2020	Does not recover sufficiently to compute recovery metrics
Hospitalizations	Louisiana	Spring 2020	
	New Jersey	Spring 2020	
	Alabama	Summer 2020	Does not recover sufficiently to compute recovery metrics
	Arizona	Summer 2020	
	California	Summer 2020	
	Colorado	Summer 2020	
	Connecticut	Summer 2020	
	Louisiana	Summer 2020	
	Massachusetts	Summer 2020	
	Nevada	Summer 2020	Does not recover sufficiently to compute recovery metrics
	South Carolina	Summer 2020	Does not recover sufficiently to compute recovery metrics
	Texas	Summer 2020	

### Ground truth data processing

The ground truth data for each peak event was processed to determine probability distributions for date of peak, magnitude of peak, and date of recovery if sufficient recovery occurred. To account for uncertainty a Bayesian calibration approach was used. We fit the following statistical model:

$$\begin{array}{c} \text{Observed}∼\text{NegativeBinomial}(\lambda (t),\alpha ) \end{array}$$

where *α* is the Negative Binomial over-dispersion parameter and the mean function λ(*t*) is the expected number of the deaths/hospitalizations at date *t* and is represented as a log-transformed spline

$$\begin{array}{c} \lambda \left(t\right)=\text{exp}(\sum_{j}^{N}w_{j}b_{j}\left(t\right)) \end{array}$$

where *b*_*j*_ are the spline bases and *w*_*j*_ the weights to be determined. This approach was also used in [39]. The following relatively uninformative priors were used: *w*_*j*_ ∼ *N*(*c*_1_, *c*_2_) and *α* ∼ Γ(2, 2), where *c*_1_ is the max of the log of the observed data divided by the number of knots, and *c*_2_ was empirically chosen as 1 for deaths and 2 for hospitalizations (2.5 for some states with larger hospitalization counts). The MCMC method was used to estimate the posterior distribution of the peak date and peak magnitude, using the Python library pymc3 which implements the No U-Turn Sampler (NUTS), a Hamiltonian MCMC algorithm [40]. The number of knots was chosen by fitting the model with variable number of knots and selecting the number which maximized the leave-one-out (LOO) cross validation score [41]. We also quantified the rate of recovery by determining probability distributions for the recovery date, defined as the date the deaths/hospitalizations first drop below a threshold of 40% of a median peak magnitude. Some peak events did not recover according to this definition, as indicated in Table 2.

### Validation scores

Next, we defined a range of validation scores that evaluate different aspects of model performance.

#### Accuracy of date of peak

One key question public health decision-maker may ask of a model is when an emerging local wave will peak. We defined three scores that assess the ability of a model to answer this question. In this paper we use publicly available model predictions which typically provide a mean prediction of deaths/hospitalizations and sometimes also lower and upper bound for each day’s deaths/hospitalizations. However, it is not possible to infer from this information uncertainty ranges for peak date predictions. In this paper we therefore only evaluate the mean predictions and do not include prediction uncertainty in our scoring. This limitation is discussed in the Discussion section.

For a given peak event, let *f*_*D*_ represent the posterior distribution for true peak date (Ground Truth Data Processing section), and let [*d*_*l*_, *d*_*u*_] represent 99% highest density interval for *f*_*D*_. We define one time-varying error and two scalar validation scores:

- PeakDate_Error: *error in the predicted peak date as a function of model release date*. Defined as *e*(*p*_*i*_, *f*_*D*_), where *p*_*i*_ is the model-predicted peak date for release *m*_*i*_, and *e* is the area metric between two cumulative distribution functions (CDFs) [42], interpreting the scalar *p*_*i*_ as a step function CDF.
- PeakDate_FirstAccurate: *days prior to peak that the model first accurately predicted the peak date*. Defined as *d*_*l*_ − d*, where d* is the release date of m*, the first release to ‘correctly’ predict a peak date ∈ [*d*_*l*_ − *τ*_*D*_, *d*_*u*_ + *τ*_*D*_] (score of zero if no such release). *τ*_*D*_ is a chosen tolerance. Only predictions released in [*d*_*l*_ − 40, *d*_*l*_] are considered, that is, releases up to 40 days before peak.
- PeakDate_FirstConsistent: *days prior to peak that the model consistently and accurately predicted peak date*. Defined as *d*_*l*_ − d*, where d* is the release date of m*, first release such that m*and all subsequent releases predict a peak date ∈ [*d*_*l*_ − *τ*_*D*_, *d*_*u*_ + *τ*_*D*_] (score of zero if no such release). *τ*_*D*_ is a chosen tolerance. Again, only models released in [*d*_*l*_ − 40, *d*_*l*_] are considered.

The tolerance *τ*_*D*_ was chosen so that each window was at least seven days in width.

#### Accuracy of magnitude of peak

Model predictions on upcoming peak magnitudes provide information on the maximum burden to be placed on the healthcare system. Let [*v*_*l*_, *v*_*u*_] be the 99% highest density interval for peak magnitude and *v*_*m*_ be the median value. Analogously to peak date, we define scores:

- PeakMagnitude_Error: *error in the predicted peak magnitude as a function of release date*. Defined as PeakDate_Error using model predicted peak magnitude and peak magnitude ground truth CDF.
- PeakMagnitude_FirstAccurate: *days prior to peak that the model first accurately predicted the peak magnitude*. Defined as PeakDate_FirstAccurate except m* is first release to predict a peak magnitude ∈ [*v*_1_ − *τ*_*M*_*v*_50_, *v*_99_ + *τ*_*M*_*v*_50_], where *τ*_*M*_ is a chosen relative tolerance.
- PeakMagnitude_FirstConsistent: *days prior to peak that the model first accurately and consistently predicted the peak magnitude*. Defined as PeakDate_FirstConsistent except m* is first release such that m* and all subsequent releases predict a peak magnitude ∈ [*v*_1_ − *τ*_*M*_*v*_50_, *v*_99_ + *τ*_*M*_*v*_50_], where *τ*_*M*_ is a chosen relative tolerance.

The tolerance *τ*_*M*_ was chosen so that each window was at least 20% of median peak magnitude in width.

#### Accuracy in identifying if peak has occurred

When a local wave is appearing to be near or at peak, models provide insight into whether the peak has occurred or is yet to come. We test the model predictive capability in that regard with two metrics.

- PeakInFuture_Accuracy: *how accurate the model was in identifying the peak was still to come, for model releases prior to the peak*. Defined as percentage of model releases which correctly identified the peak has not yet occurred, from all the releases (including dummy releases) ∈ [*d*_*l*_ − 40, *d*_*l*_].
- PeakInPast_Accuracy: *how accurate the model was in identifying the peak has passed, for models releases just after the peak*. Defined as percentage of model releases which correctly identified the peak has passed, from all releases (including dummy releases) ∈ [*d*_*u*_, *d*_*u*_ + 14].

#### Accuracy of total number of deaths in a fixed period

Understanding the total number of deaths or hospitalizations that will occur in the near future is important in predicting overall demand for personnel, PPE and medical products. To assess the quality of these predictions, we defined ‘future cumulative deaths’ as the total number of deaths occurring in 28 days from a model release date. For a given release on day *d*_*i*_, we compute the relative error in future cumulative deaths, *e*_*i*_ = |*p*_*i*_ − *o*_*i*_|/*o*_*i*_, where *p*_*i*_ is the predicted value from the model release that day and *o*_*i*_ is the true total number of deaths over the next 28 days. We then define the following score:

- Cumulative_AverageError: *average relative error in future cumulative deaths over all releases considered*. Defined as ∑ |*e*_*i*_|/*n*, summing over all releases in [*d*_*l*_ − 40, *d*_*u*_], i.e., before and during peak.

#### Accuracy of recovery prediction

Model predictions on the length of the recovery period provide information on how long public health measures may need to be in place for. We characterized accuracy of recovery predictions as follows. As discussed in the Ground Truth Data Processing section we defined, for each peak event, a ‘recovery date’ as the date that deaths/hospitalizations fell below a threshold value of 40% of peak (specifically, 40% of median peak magnitude). Recovery date is a surrogate for rate of recovery. Each release was analyzed to identify model-predicted recovery date, i.e., the date the model release predicts deaths/hospitalizations will drop below same threshold. We then computed

- RecoveryDate_Error, *error in the predicted recovery date as a function of release date*. Defined analogously to PeakDate_Error, using model predicted date of recovery and recovery date CDF.

We did not define recovery equivalents to PeakDate_FirstAccurate, PeakDate_FirstConsistent for reasons to be discussed in the Recovery Predictions section in Results.

## Results

### Processing of ground truth datasets

**Fig 3:**
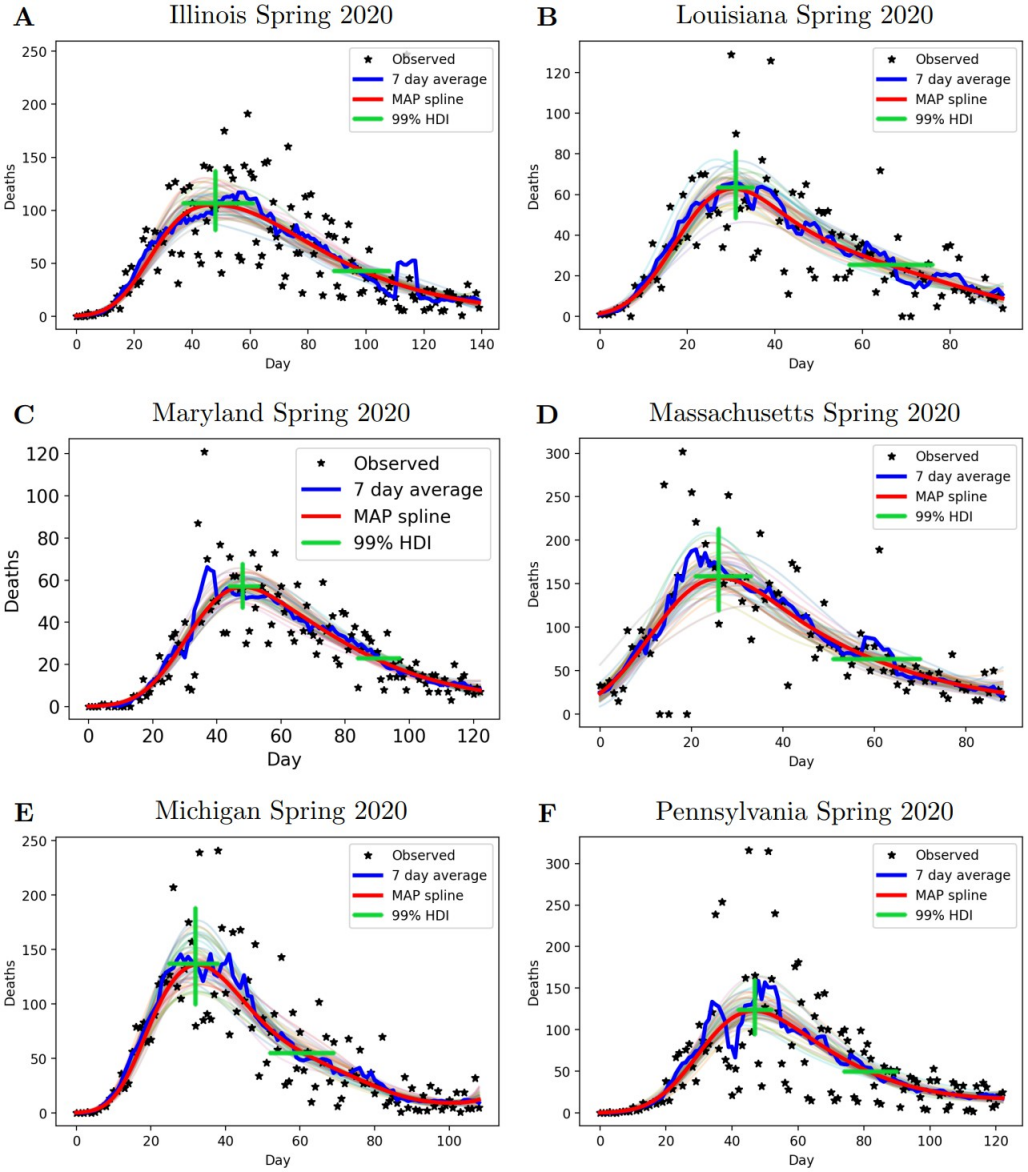
Statistical fits for all Spring 2020 death peak events. A) Illinois, B) Louisiana, C) Maryland, D) Massachusetts, E) Michigan, and F) Pennsylvania. The red spline corresponds to maximum *a posteriori* parameters, the blue line is the 7-day rolling average, and the green lines are the 99% highest density intervals for peak date, peak magnitude and recovery date (recovery to 40% of peak). The various translucent colored lines in the background represent splines sampled from the posterior distribution.

Fig 3 presents the results of the statistical fits for the six death peak events that occurred in Spring 2020. The statistical fits for the remaining four death peak events that occurred in Summer 2020, and all 12 hospitalization peak events, are provided in Section 1 of the S1 Text. The 99% highest density intervals for the peak date, peak magnitude and recovery date are indicated using green lines. We observe reasonable uncertainty bounds that align with what might be expected from observing the seven-day rolling average. This indicates a robust method to determine the uncertainty bounds for the date of peak and the magnitude of peak for each peak event.

### Model accuracy in predicting date and magnitude of peak deaths

**Fig 4:**
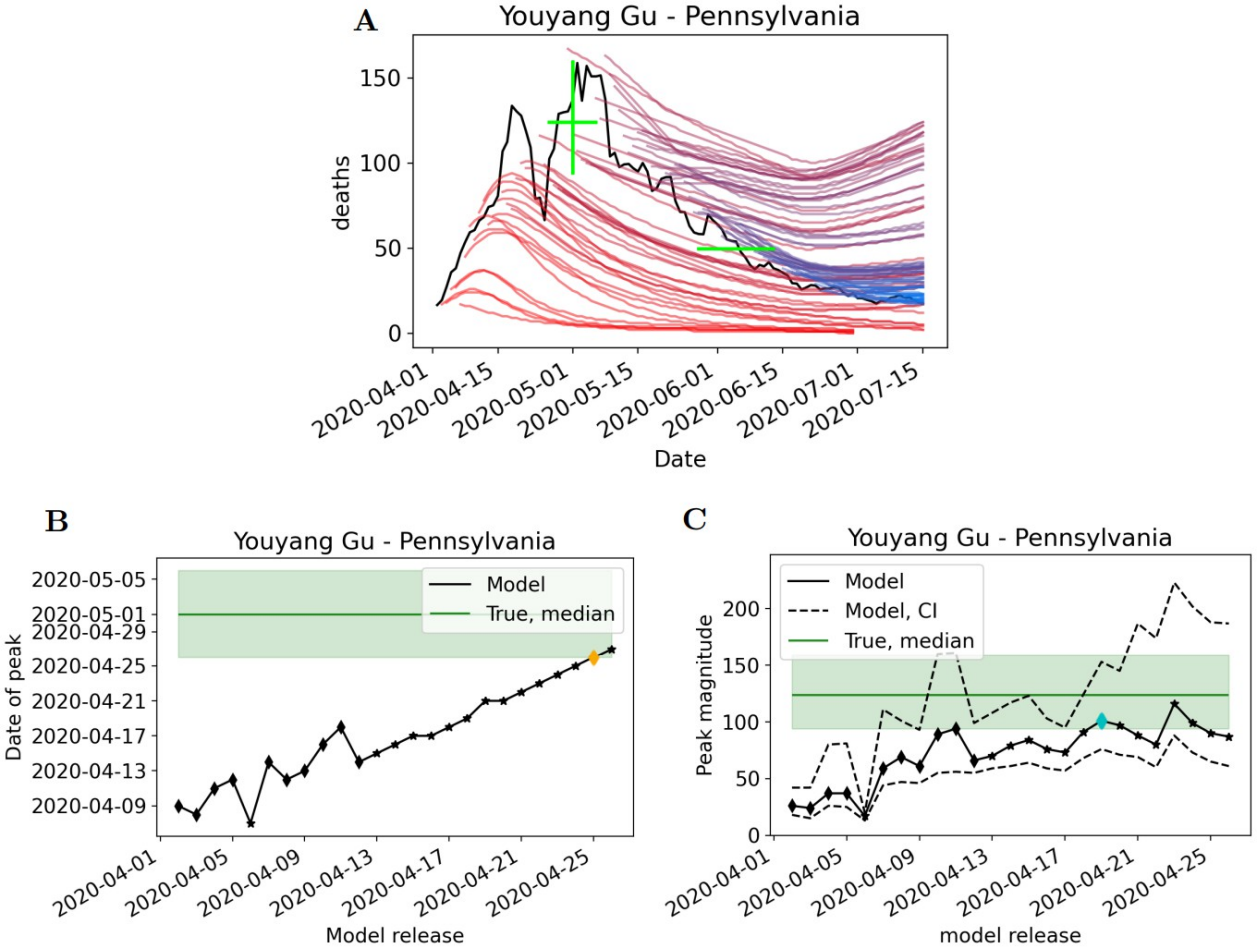
Multi-release and ‘convergence plots’ of the YYG forecast of Pennsylvania’s peak during Spring 2020. A) The multi-release plot demonstrates the YYG forecasts from April 2020 through July 2020. Red lines indicate early model releases; blue lines indicate later model releases; purple are intermediate. Green lines represent uncertainty in true peak date/magnitude and black line is the seven-day rolling average. B) The convergence plot for peak date demonstrates the uncertainty in true peak date (green shaded region; median provided for reference) and the YYG predictions of the peak date for each model release leading up to the peak. C) Corresponding plot for peak magnitude. The blue diamond represents first release the prediction was inside the green window (PeakDate_FirstAccurate) and the orange diamond represents the model is consistently inside the window of peak date/magnitude from that release onwards (PeakDate_FirstConsistent).

Fig 4 provides an example of all model releases (YYG model) during a peak event (Pennsylvania Spring 2020 deaths), along with the corresponding plots of predicted peak date and peak magnitude for each release. Convergence of model predictions towards the true value, or lack thereof, can be seen, and the releases corresponding to PeakDate_FirstAccurate and PeakDate_FirstConsistent are highlighted. This is illustrative of the process performed on each model for all the peak events. Corresponding figures for the other models are provided in Section 2 of the S1 Text. Multi-release figures for every model and every peak event are provided in Section 3 of the S1 Text.

**Fig 5:**
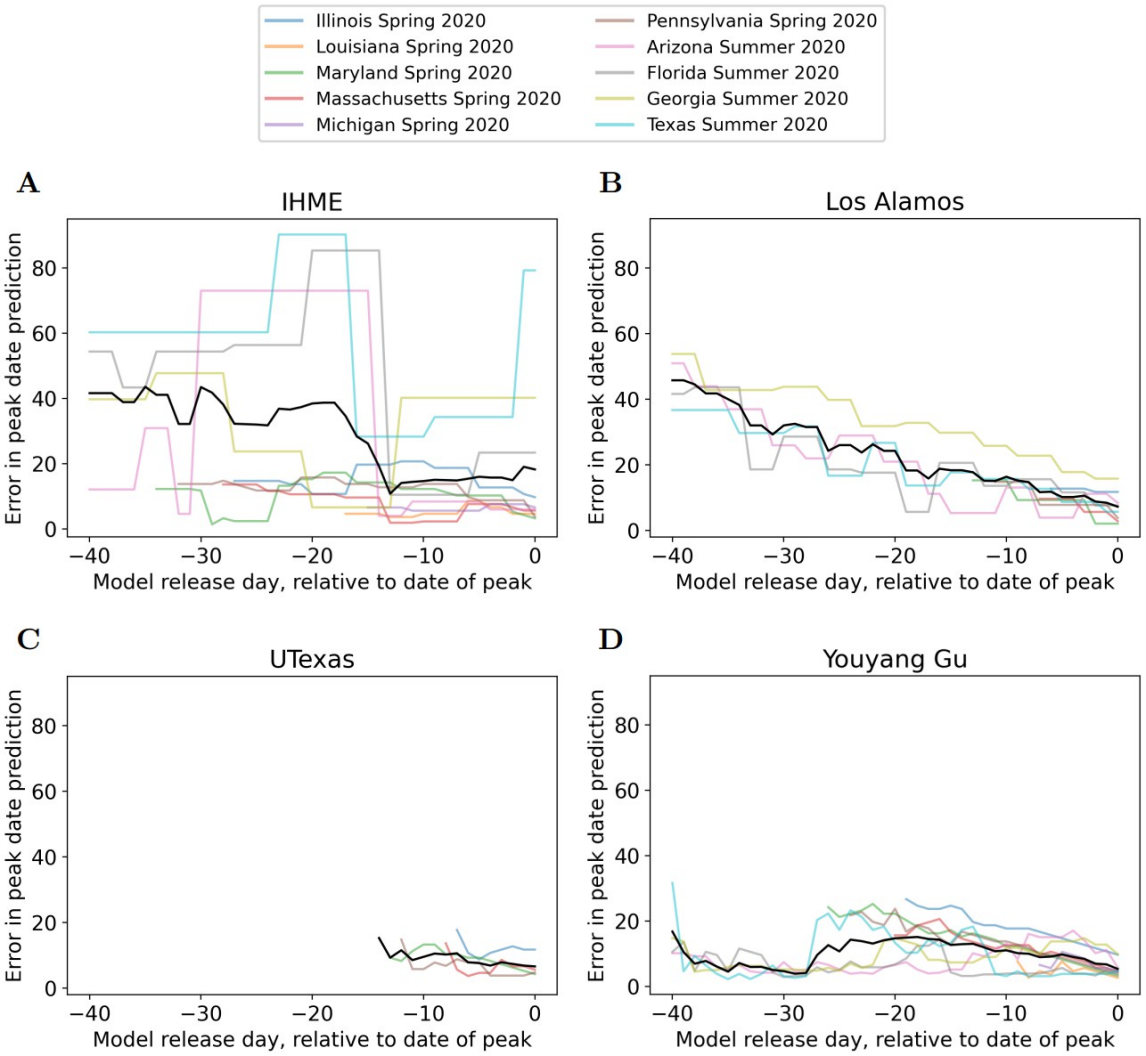
Errors in prediction of date of peak deaths. A) IHME, B) Los Alamos, C) UTexas, and D) YYG. Colored lines represent different peak events, black line is average across peak events. Lines that begin later than -40 begin on date of first model release. If the model did not predict a peak would occur the error is undefined so no line is plotted.

**Fig 6:**
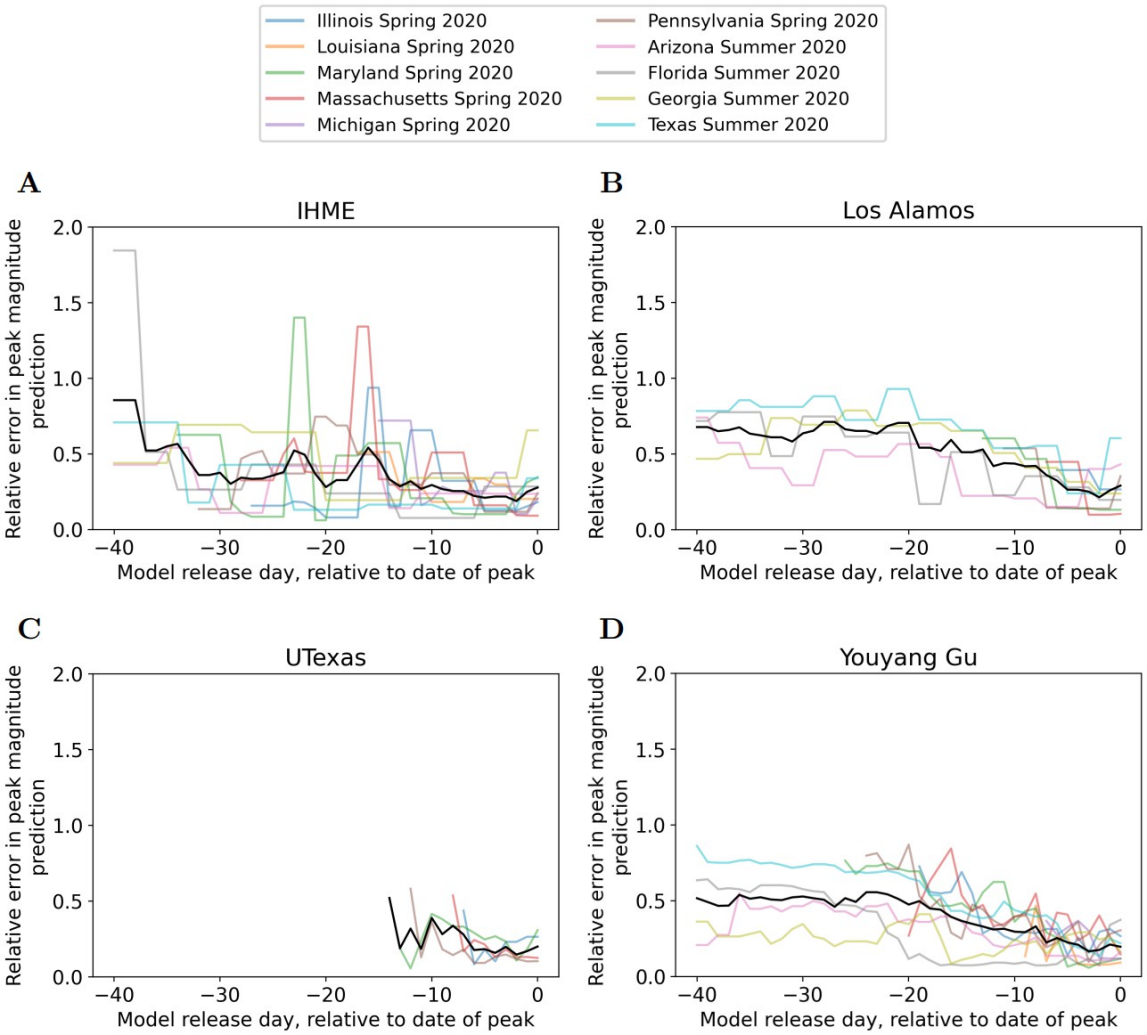
Errors in prediction of magnitude of peak deaths. A) IHME, B) Los Alamos, C) UTexas, and D) YYG. Colored lines represent different peak events, black line is average across peak events. Lines that begin later than -40 begin on date of first model release. If the model did not predict a peak would occur the error is undefined so no line is plotted.

Figs 5 and 6 plot the errors in prediction of date of peak deaths, and magnitude of peak deaths, respectively. These figures plot PeakDate_Error and PeakMagnitude_Error for all peak events, together with their average over peak events. Note that for the four summer 2020 peak events UTexas did not predict any peak occurring. This meant that the error in predicting peak date/magnitude was undefined. Therefore the four Summer 2020 peak events were not included in Figs 5 and 6 and hence there were less lines for UTexas than other models. (These lines start later than −40 because the first model release for these spring events was only shortly before the peak occurred).

Fig 5 demonstrates large variability in peak date accuracy across the peak events. Some models were more variable than others—e.g. IHME had larger variability than the others; Los Alamos was quite consistent across peak events. Looking at the average error across peak events for date of peak, the YYG model exhibits the best performance. This includes impressive accuracy several weeks before the peak occurs, as this model generally had a 10–15 day error in date of peak over a month before the peak occurs.

Fig 6 also demonstrates large variability in peak magnitude accuracy across the peak events. Looking at the average relative error, all models demonstrate relative error decreasing to approximately 20–25% as approaching the date of peak. Again, the YYG model demonstrates the best performance when excluding UTexas for limited predictions.

**Fig 7:**
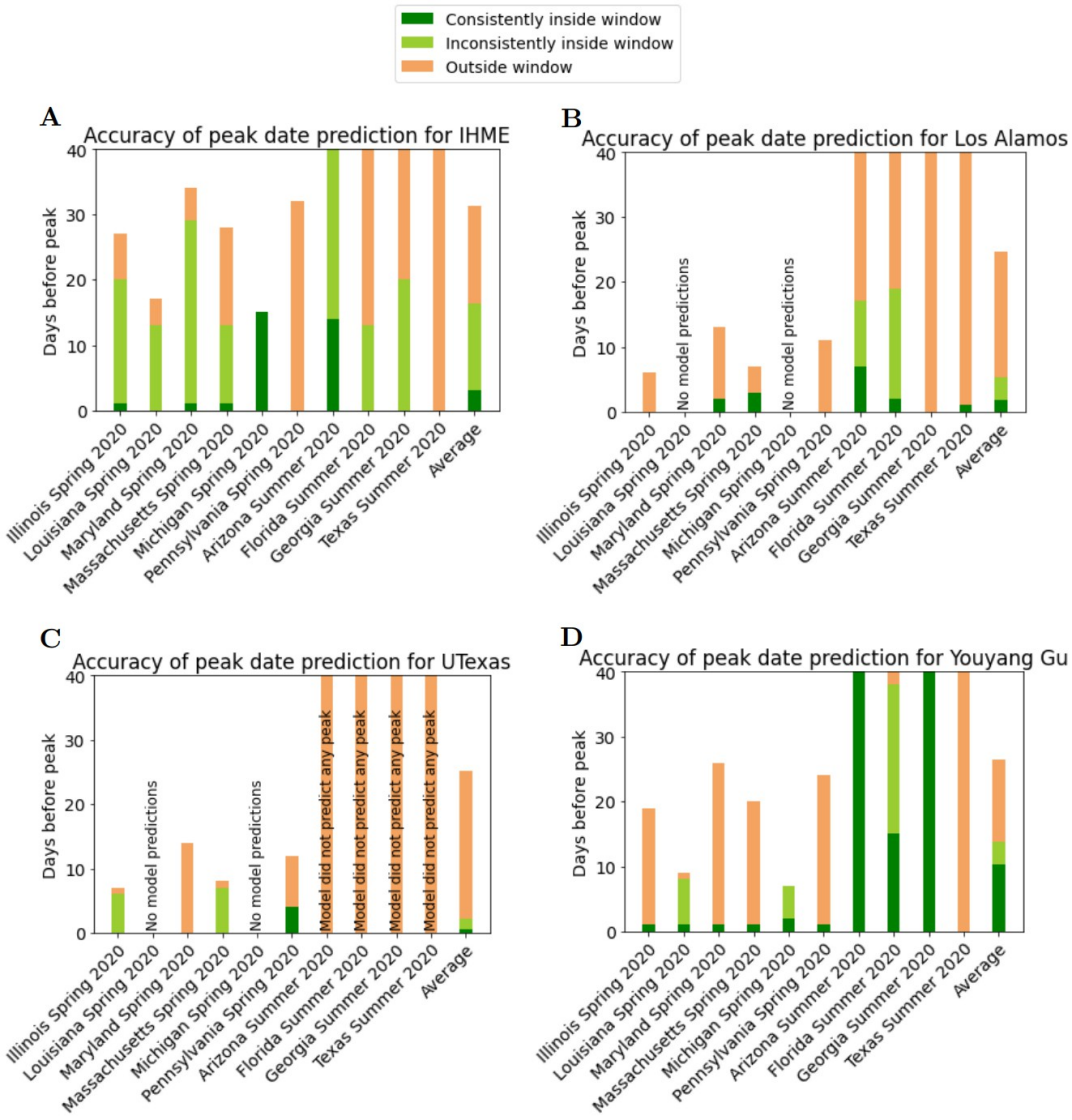
Visualization of predictive accuracy for date of peak deaths of all Spring and Summer 2020 death peak events (see Table 2). A) IHME, B) Los Alamos, C) UTexas, and D) YYG. Each bar represents a peak event. Dark green region represents the days prior to peak that the model accurately and consistently predicted date of peak (see definition of PeakDate_FirstConsistent score). Orange/light green boundary represents first day model predicted date of peak (see definition of PeakDate_FirstAccurate score).

**Fig 8:**
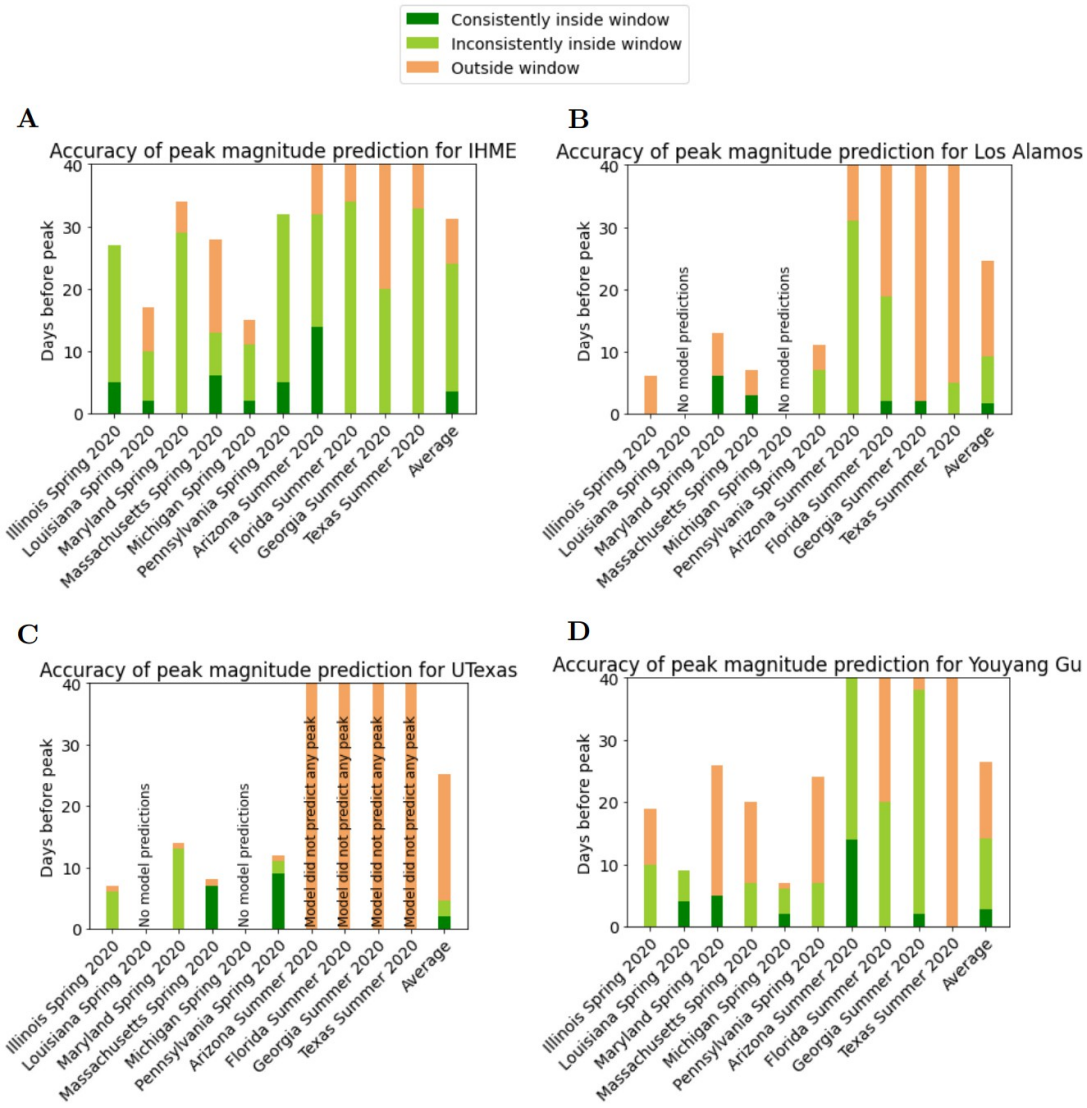
Visualization of predictive accuracy for magnitude of peak deaths of all Spring and Summer 2020 death peak events (see Table 2). A) IHME, B) Los Alamos, C) UTexas, and D) YYG. Each bar represents a peak event. Dark green region represents the days prior to peak that the model accurately and consistently predicted magnitude of peak (see definition of PeakMagnitude_FirstConsistent score). Orange/light green boundary represents first day model predicted magnitude of peak (see definition of PeakMagnitude_FirstAccurate score).

Figs 7 and 8 complement Figs 5 and 6. These figures are based on the computed values of PeakDate_FirstAccurate and PeakDate_FirstConsistent. They provide information on how far in advance of a peak models consistently and accurately predict date/magnitude of peak (with ‘accurate’ defined as being within a given window).

Considering Fig 7 first, again there is substantial variability in model performance across peak events. However, for YYG there is substantial dark green, indicating consistent accuracy, during the summer peak events. The YYG model prediction for date of peak is consistently accurate from 10 days before the peak, on average, due to exceptional performance on two states—Arizona and Georgia. The YYG peak date predictions noticeably improve for the summer events over the spring events—this is the only case of a model showing increased accuracy later in the pandemic for the results in this paper. Although this improvement of performance over time may be location dependent, in general YYG’s performance of peak events early in the pandemic did not perform as well compared to later peak events. The IHME model has substantial light green, indicating that the model identifies an accurate peak date well in advance of the peak date, but was inconsistent in subsequent releases. Overall, only the model YYG exhibited consistent accuracy in advance of peak.

In the corresponding results for peak magnitude (Fig 8), no model exhibited appreciable consistent accuracy. IHME has the best performance with substantial light green, indicating that the model again identifies an accurate magnitude of peak well in advance of the peak date, however is inconsistent for subsequent releases. Overall, each model on average predicts the magnitude of peak only a few days before the peak. Therefore, these models are inconsistently accurate at best.

From Figs 7 and 8 with the exception of YYG, each model has a similar performance for predicting date of peak as it does predicting magnitude of peak.

### Model accuracy in predicting date and magnitude of peak hospitalizations

**Fig 9:**
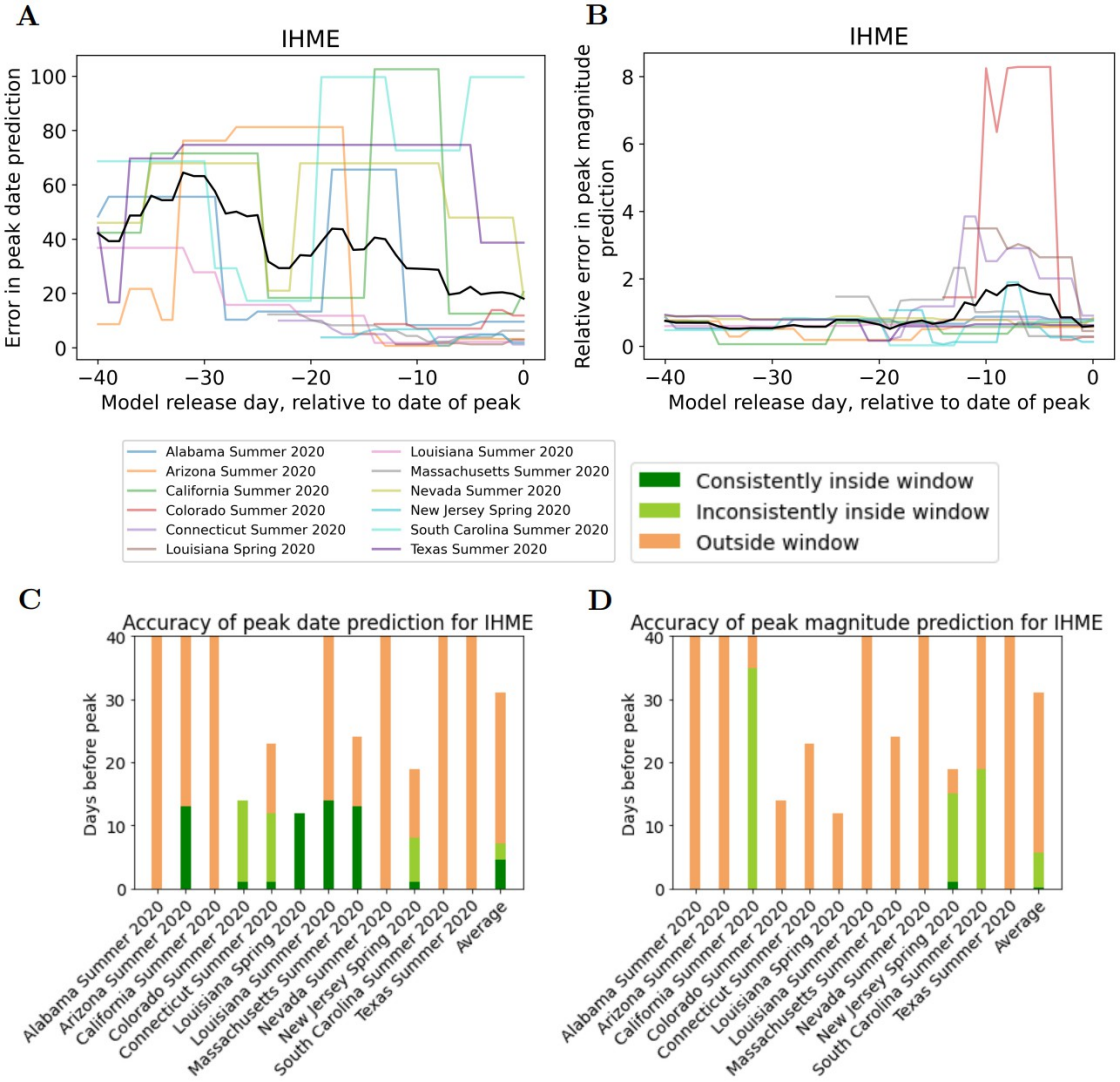
A) Errors in prediction of **date of peak hospitalizations**. B) Errors in prediction of **magnitude of peak hospitalizations**. Colored lines represent different peak events, black line is average across peak events. Lines that begin later than -40 begin on date of first model release. C) Visualization of predictive accuracy for **date of peak hospitalizations** of all Spring and Summer 2020 hospitalization peak events (see Table 2). D) Visualization of predictive accuracy for **magnitude of peak hospitalizations** of all Spring and Summer 2020 hospitalization peak events (see Table 2). Each bar represents a peak event. Dark green region represents the days prior to peak that the model accurately and consistently predicted date of peak (see definition of PeakDate_FirstConsistent score). Orange/light green boundary represents first day model predicted date of peak (see definition of PeakDate_FirstAccurate score).

Corresponding figures of predictions of peak date and magnitude for hospitalizations are provided in Fig 9. As discussed in the Data Sources and Models section, the only model to be assessed is the IHME model. Fig 9 provides the error for each peak event and average error, together with information on predictive accuracy and consistency.

We observe again that there is substantial variability in model performance across peak events. We also notice that IHME model peak date error for hospitalizations is similar to peak date error for deaths. However, the IHME model peak magnitude error for hospitalizations is greater than peak magnitude error for deaths. Looking at the average errors, there is a less of a decreasing trend for hospitalization predictions than for the death predictions.

### Other metrics

Values of additional validation scores are presented in Table 3 in which we analyze Cumulative_AverageError, PeakInFuture_Accuracy, and PeakInPast_Accuracy for each model. Reported are the means across peak events and standard deviations. We observe large standard deviations throughout the table. This indicates immense variability in model performance across peak events. We notice that the YYG model performed the best in predicting cumulative deaths over the next month. All models except IHME were much better at determining that the peak has passed rather than the peak had not yet occurred (deaths). Curiously, for hospitalizations, IHME was better at determining that peak was yet to come rather than it had passed.

**Table 3:** Values of all other validation metrics. Mean ± SD are presented, where the mean is average of metric across the peak events. n = number of peak events.

	Metric	IHME	YYG	UTexas	Los Alamos
Deaths	Cumulative_AverageError	0.42 ± 0.12 (n = 10)	0.33 ± 0.12 (n = 10)	0.65 ± 0.13 (n = 8)	0.38 ± 0.11 (n = 8)
	PeakInFuture_Accuracy	81% ± 13% (n = 10)	66% ± 26% (n = 10)	52% ± 24% (n = 4)	38% ± 29% (n = 8)
	PeakInPast_Accuracy	79% ± 34% (n = 10)	90% ± 28% (n = 10)	91% ± 9% (n = 4)	100% ± 0% (n = 8)
Hospitalization	Cumulative_AverageError	0.89 ± 0.37 (n = 12)			
	PeakInFuture_Accuracy	86% ± 8% (n = 12)			
	PeakInPast_Accuracy	49% ± 47% (n = 12)			

### Recovery predictions

**Fig 10:**
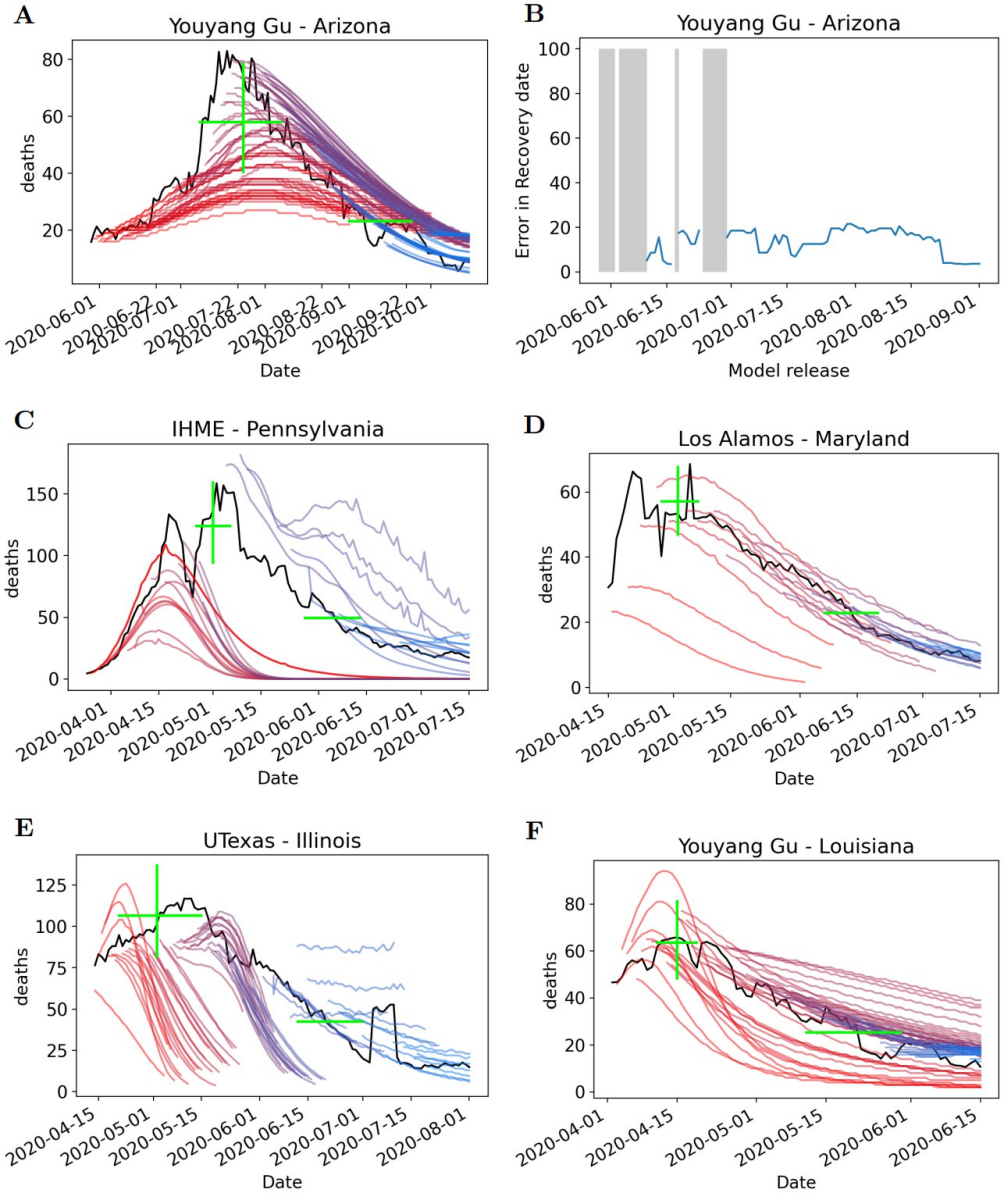
Visualization of recovery predictions for various peak events of four different models. Shaded grey region(s) indicate no date of recovery was predicted. A) The Arizona Summer 2020 peak YYG’s multi-release predictions are shown along with B) its error in prediction of recovery date. C–F) Example multi-release plots for other models.

Figures presenting all predictions from every model release, for all peak events, are provided in Section 3 of the S1 Text, together with recovery error plots for peak events for which recovery (according to the definition in the Ground Truth Data Processing section) occurred. Some sample figures are provided in Fig 10. Recall that we assess the performance of recovery based on a recovery date which is a surrogate for the rate of recovery. Unlike for date of peak we have not averaged errors across peak events, i.e., there are no equivalent figures to Fig 5. This is because there were many model releases for which no date of recovery was predicted (e.g., because the release predicted a plateau or limited recovery), and therefore the error is undefined. This is indicated by the shaded grey regions in Fig 10B. Therefore, our analysis is based on qualitative inspection of multiple figures. Also, we did not compute quantities such as RecoveryDate_FirstAccurate or RecoveryDate_FirstConsistent because there is potential for such quantities to be misleading if an early release did not accurately predict peak date or magnitude but happened to pass the recovery threshold within the correct window; see for example Fig 10A and 10B for which early releases under-estimated peak magnitude and under-estimated rate of recovery but overall accurately predicted recovery date.

Each model that has daily death predictions varies in their recovery performance. When analyzing IHME we observe improvement in recovery predictions with later releases, see for example the Spring 2020 death peak events for Illinois, Louisiana, Michigan and Pennsylvania (in Fig 10C–10F). This is likely due to the methodological change in the model. From March 25, 2020 through April 29 a statistical curve fit model was utilized. On May 4th, 2020, IHME switched to using a hybrid model, drawing on a statistical curve fit for the first stage followed by an epidemiological model with susceptible, exposed, infectious, recovered compartments for the second stage. This model was used through May 26th, 2020. After May 29th, 2020, a spline fit to the first stage was replaced by a relationship between log cumulative deaths and log cumulative cases, while the second stage remained. These methodological updates likely improved its recovery predictions.

The Los Alamos model performed better at predicting the recovery date as the releases became closer to the true recovery date (Fig 10D and Fig H–J in S1 Text). How well Los Alamos performed in predicting recovery was largely dependent on the peak event. However, overall Los Alamos did well at following the rate of recovery. UTexas demonstrated much variability in its recovery date predictions varying greatly for each peak event, including regularly predicting an overly fast rate of recovery (e.g., Fig K(A) and K(D) in S1 Text). An important note is that UTexas had a large gap in predictions for summer peak events and therefore did not have recovery predictions (Fig M in S1 Text).

The YYG model performed exceptionally well compared to the other models when predicting the recovery date for some peak events (Fig N–P in S1 Text). It was the only model that reached near-zero error in recovery date well in advance of the true recovery date. Specifically impressive performance from YYG model include the following peak events for which there was low error weeks in advance: Louisiana and Massachusetts Spring 2020, and Arizona Summer 2020 (Fig N(C), O(A), and P(A) in S1 Text). However, for several peak events: Illinois and Maryland Spring 2020, Pennsylvania Summer 2020, the YYG model predicts no recovery as the true recovery date approaches (Fig N(A), N(E), and O(E) in S1 Text).

## Discussion

### Findings for COVID-19 models

We implemented a framework to retrospectively evaluate the predictive performance of four major COVID-19 models. Performance varied greatly across models as well as the peak events analyzed. Among the four models, the YYG model exhibited the best performance, including errors in deaths peak-date prediction of around 15 days or less, for releases 3–6 weeks in advance of the peak. Furthermore, the YYG model was arguably also most successful at predicting the peak magnitude before the peak occurred, although the four models were more similar for magnitude than for date. Death peak magnitude relative errors were generally in the 50% range 3–6 weeks before peak. The IHME model was the only model we analyzed that predicted both daily deaths as well as hospitalizations. Death predictions were more accurate than hospitalization predictions, however. In general, the models were more reliable for identifying peaks had occurred vs yet to occur. Accuracy of rate of recovery was extremely variable across models and peak events. There was only one case of a model showing increased accuracy later in the pandemic (YYG model peak date predictions).

Due to the waning or transitioning modeling efforts of the teams behind the four COVID-19 models, we were limited in analyzing Spring 2020 and Summer 2020 peak events. The YYG model ceased their death forecasts after October 2020 and transitioned its modeling efforts to focus on infection estimates and vaccination projections through March 2021. The UTexas model discontinued their mortality forecast dashboard in April 2021 to focus on their other dashboards that provide a variety of COVID-19 risk assessment tools and healthcare forecasts, and provided decreased predictions in the preceding months. The Los Alamos model made its last real-time forecast in September 2021, citing that the COVID-19 forecasting community is stronger than ever. As of May 2022 the IHME model is the only model of the four we analyzed that continues to release predictions. As a result of the modeling efforts ending their predictive efforts of deaths and hospitalizations at various times we were unable to compare the predictive performance of the models in later peak events where performance could have improved. Additionally, we were only able to analyze one model which had the capabilities to predict COVID-19 hospitalizations, the IHME model. Many COVID-19 models that had hospitalization prediction capabilities did not have publicly accessible forecasts or did not cover multiple regions. As a result, we were unable to compare how IHME performed to other hospitalization predictive models. Rather, we were able to compare how well the IHME model performed when predicting hospitalizations to predicting deaths. This is limiting as it is unclear whether a model’s hospitalization predictive performance is truly comparable to a model’s death predictive performance. The performance of hospitalization predictive models may differ.

### Utility, limitations, and extensions of framework

We have provided a framework that allows for the characterization of error in quantities of interest that are relevant to end users. We base our validation metrics upon questions the models could be used to answer such as when a peak will occur, what will be the magnitude of the peak, and questions revolving around recovery. Our framework systematically handles multiple model releases and allows for comparison between models with different prediction release schedules. There is currently one other paper available, Friedman et al. [31], that addresses the predictive performance of various COVID-19 model and accounts for multiple version releases. In our framework we used a statistical model to account for the uncertainty in the date of the peak, whereas Friedman et al. [31] utilized a loess (locally weighted smoothing) filter. Additionally, their framework examines the predictive performance of models covering various countries. Our framework differs as we examine peak events across all of the US states satisfying our inclusion criteria during Spring and Summer 2020. Furthermore, we examined accuracy of magnitude of peak and recovery rate, not considered in Friedman et al. [31].

The framework we developed is unique. Rather than focusing on global statistical error metrics related to the performance of a model, we have centered our metrics on values that would answer specific questions, such as how far off was the model in predicting the peak date or peak magnitude. This allows for the end user to easily evaluate how well the model performed without the need for understanding statistical measures. One can evaluate multiple models with this framework to evaluate how under different circumstances (e.g. timing of peak event, deaths/hospitalizations) a model may perform better or worse. As a result, the user has a more comprehensive understanding on the performance of a model. This in turn can be utilized in future public health emergencies as models that were developed in the past for similar circumstances could be utilized with the understanding of what the model performs well at predicting and not predicting.

In our framework, there are limitations in its structure and ability. This framework is for retrospective evaluation of the predictive performance of models. In our instance of evaluating COVID-19 models, this means that a peak event must have already occurred. Thus, this framework cannot be utilized in determining how well a model will perform in the early stages of a future epidemic/pandemic. However, this framework can provide evidence of a model’s performance for models of future diseases if the modeling framework is similar. Furthermore, our framework does not account for the model’s confidence intervals. Thus our framework is not accounting for the model’s uncertainty in predicting the date of peak, magnitude of peak, or recovery. Additionally, we utilized an area metric to calculate the error in quantities of interest. However, the area metric can never be zero when comparing deterministic model prediction with the uncertain ground truth.

There are also some manual components in the arrangement of our analysis. We have semi-subjective criteria for identifying peak events in which we observe the data visually and make a decision. Additionally, there is some manual effort necessary when setting up and running the statistical model, which has to be performed for each peak event. Furthermore, our recovery is semi-qualitative in nature.

Finally, one unsatisfactory aspect of the framework is the arbitrary nature of which releases are included when calculating scores (e.g., choices such as *d*_*l*_ − 40 in the Validation Scores section).

Our framework could be extended to provide further information relevant to model-using decision makers. One option is to alter the error metrics to no longer treat under-estimation and over-estimation symmetrically, since for epidemiological models under-estimation has very different real-world consequences compared to over-estimation, especially when predicting peak magnitudes. Another important possible validation score is to quantify how often a model correctly identifies that a state will fail to recover after a peak. This would require defining criteria for, and then identifying, ‘plateau events’ instead of peak events, and then for example quantifying how early a model correctly predicts that deaths/hospitalizations will plateau at a high level rather than recover.

### Outlook and closing thoughts

Disappointingly, few models provided all previous forecasts, so we could not perform this analysis on most models. Many models did not provide public access that is inclusive of all of their data, with both previous and current predictions. Thus, we hope this work along with Friedman et al. [31] encourages future efforts of modelers to make all of their forecasts available.

Ultimately, we are interested in models of PPE and device demand. This framework will be used as the foundation of future efforts in evaluating the predictive performance of PPE and device demand models. Unfortunately, few models provided hospitalization predictions but our results demonstrate that there is large uncertainty in the accuracy of hospitalization predictions that can form the basis of demand predictions.

Overall, our framework provides a wealth of information about the predictive accuracy of epidemiological models, that are more easily interpretable than global error metrics such as MAE/MAPE, likelihoods or WIS, although we recommend that this framework be used in conjecture with methods computing such global metrics. We believe this framework can therefore serve as a powerful tool in future public health emergencies, either through evaluation of new mathematical models as the epidemic occurs, or through supporting the use of existing modeling methodologies for the new epidemic.

## Supporting information

S1 Text Supplementary material. The supplementary material document contains statistical fits for peak events not shown here, example convergence plots for models not shown here, and all model predictions for all death and hospitalization peak events, together with recovery error plots.
(PDF)
