# Supplementary material for: Validation framework for epidemiological models with application to COVID-19 models
Source: PLoS Comput Biol. 2023 Mar 29;19(3):e1010968. doi: 10.1371/journal.pcbi.1010968 (PMC10057797; doi:10.1371/journal.pcbi.1010968)
Supplement: S1 Text — The supplementary material document contains statistical fits for peak events not shown here, example convergence plots for models not shown here, and all model predictions for all death and hospitalization peak events, together with recovery error plots. (PDF) [file pcbi.1010968.s001.pdf]

Supplemental Material for: Validation framework for epidemiological models with application to COVID-19 models

Kimberly A. Dautel, Ephraim Agyingi, and Pras Pathmanathan

1 Remaining results of processing ground truth datasets

The results of the statistical fits are provided in Section 1 of the Supplement illustrating Summer 2020 peak events for deaths and all peak events for hospitalizations.

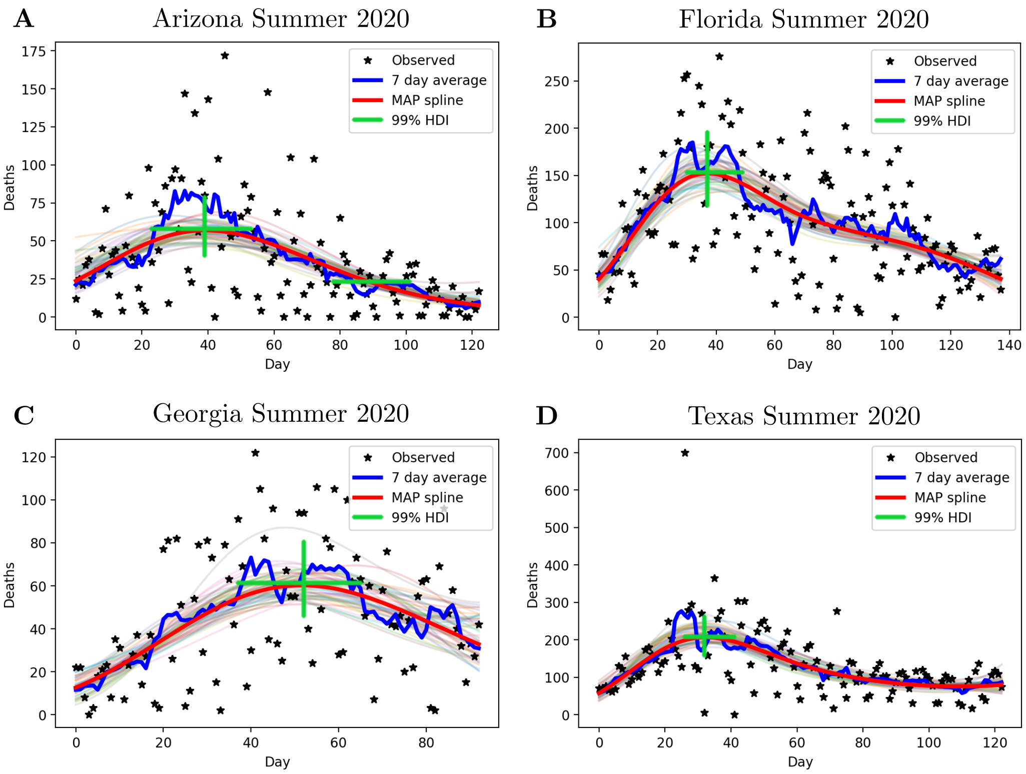

Figure A: Statistical fits for Arizona (subfig A), Florida (subfig B), Georgia (subfig C), and Texas (subfig D) during Summer 2020 death peak events. The red spline corresponds to maximum *a posteriori* parameters, the various translucent colored lines in the background represents splines sampled from the posterior distribution, the blue line is the 7-day rolling average, and the green lines are the 99% highest density intervals for peak date, peak magnitude and recovery date (recovery to 40% of peak).

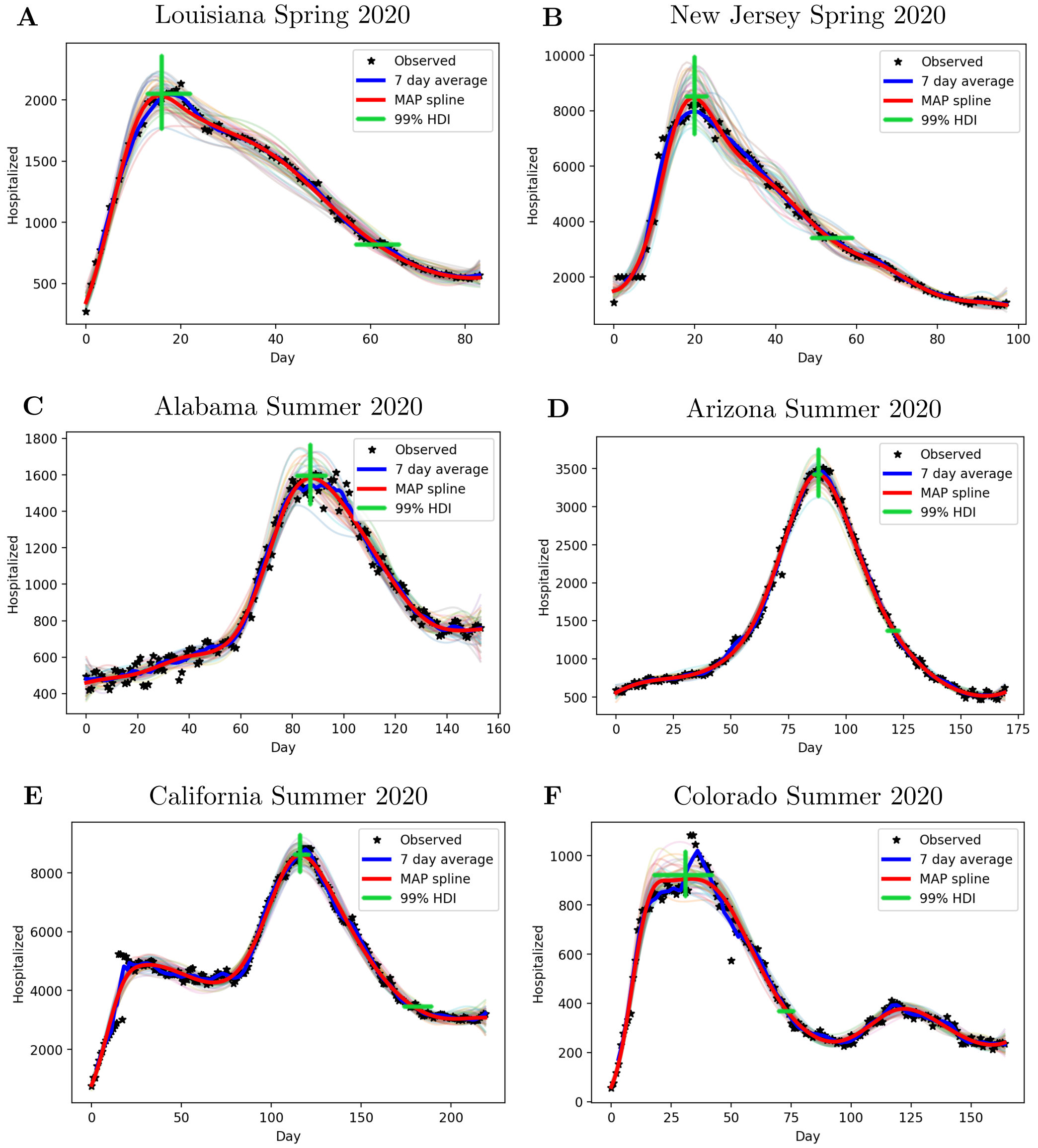

Figure B: Statistical fits for Louisiana (subfig A) and New Jersey (subfig B) during the Spring 2020 and Alabama (subfig C), Arizona (subfig D), California (subfig E), and Colorado (subfig F) during Summer 2020 hospitalization peak events. The red spline corresponds to maximum *a posteriori* parameters, the various translucent colored lines in the background represents splines sampled from the posterior distribution, the blue line is the 7-day rolling average, and the green lines are the 99% highest density intervals for peak date, peak magnitude and recovery date (recovery to 40% of peak).

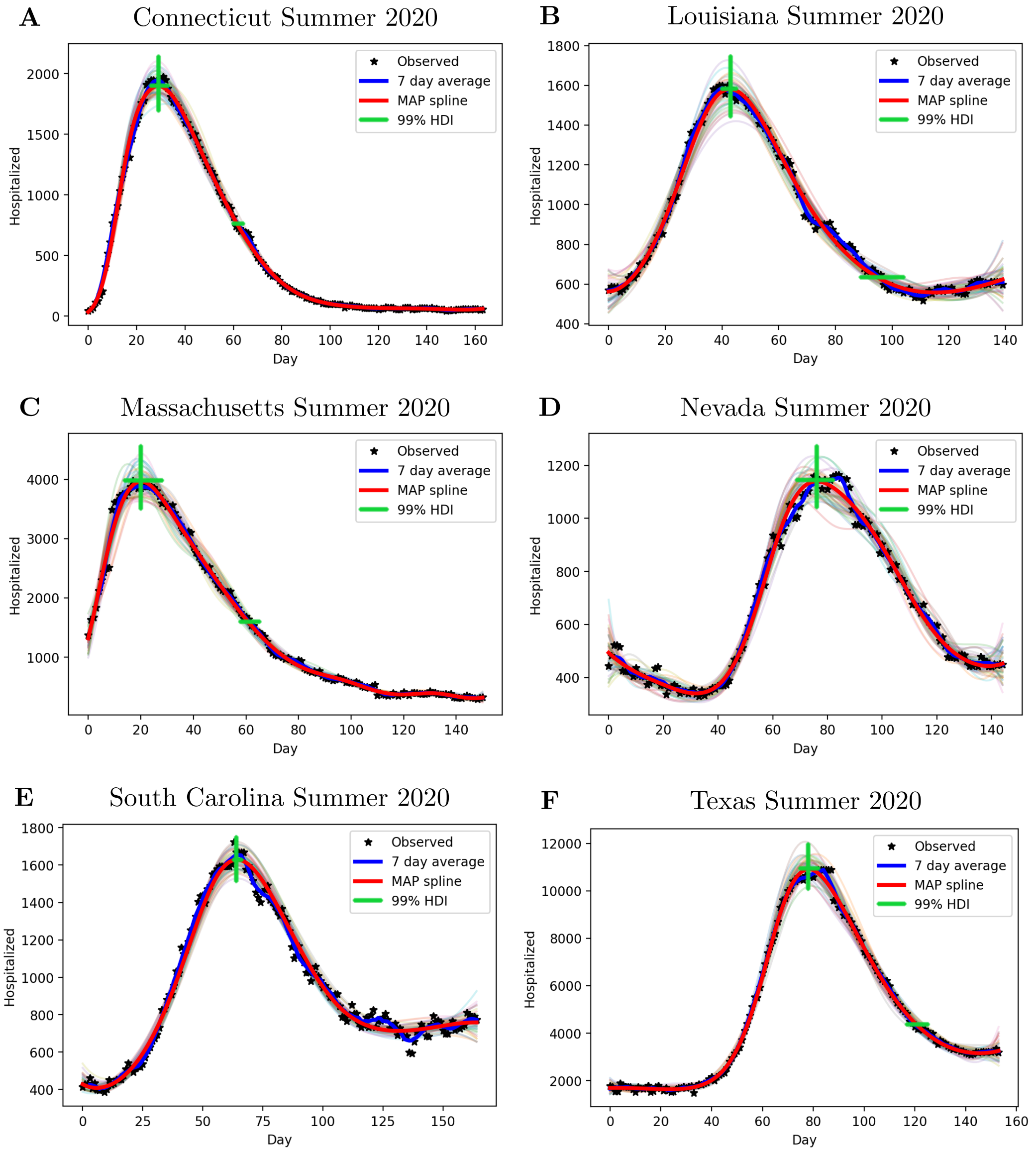

Figure C: Statistical fits for Connecticut (subfig A), Louisiana (subfig B), Massachusetts (subfig C), Nevada (subfig D), South Carolina (subfig E), and Texas (subfig F) during Summer 2020 hospitalization peak events. The red spline corresponds to maximum *a posteriori* parameters, the various translucent colored lines in the background represents splines sampled from the posterior distribution, the blue line is the 7-day rolling average, and the green lines are the 99% highest density intervals for peak date, peak magnitude and recovery date (recovery to 40% of peak).

2 Example convergence plots

Section 2 of the Supplement provides an example of all model releases during the Pennsylvania Spring 2020 deaths peak for the IHME, Los Alamos, and UTexas model, along with the corresponding plots of predicted peak date and peak magnitude for each release.

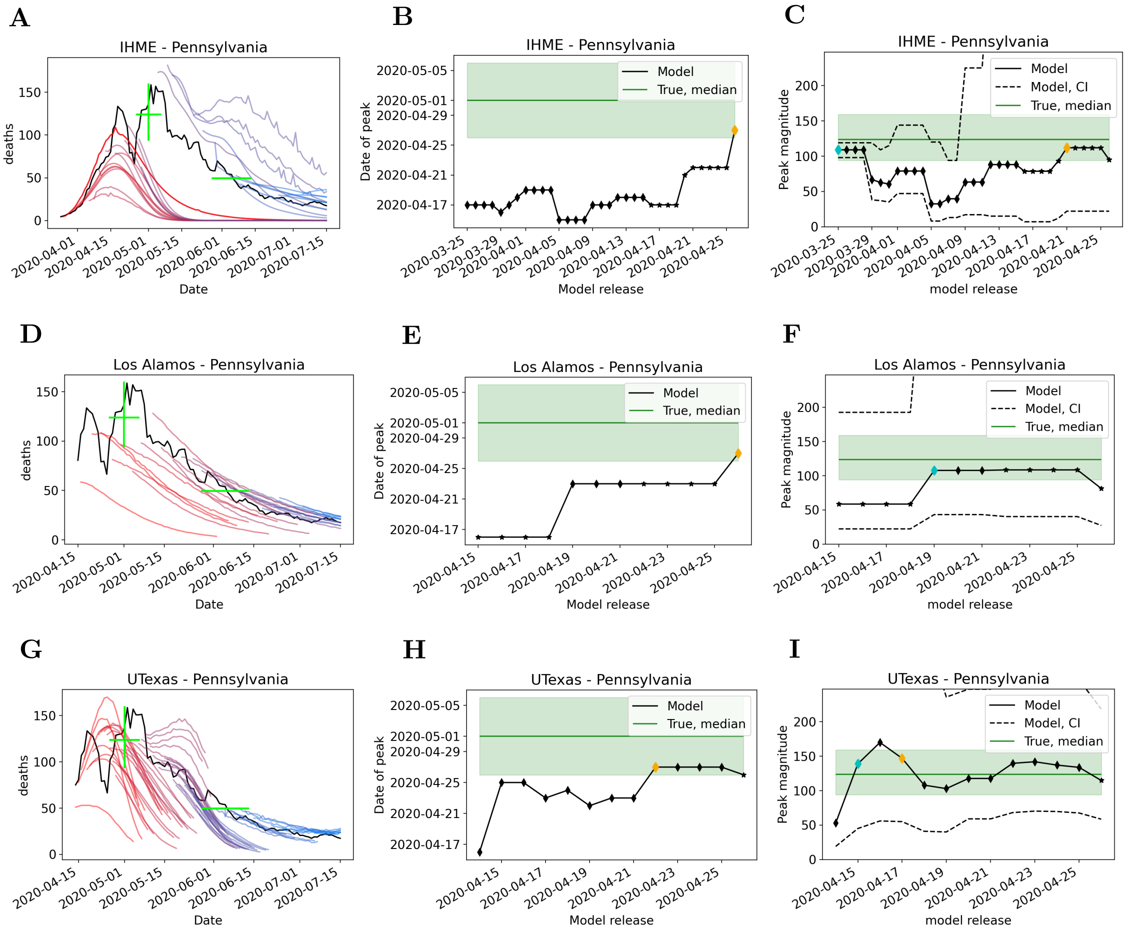

Figure D: Multi-release and convergence plots of IHME, Los Alamos, and UTexas' forecast of Pennsylvania's peak during the Spring 2020. The multi-release plot (subfigs A, D, and G) demonstrates the model's updated forecasts from April 2020 through July 2020. Red lines indicate early model releases; blue lines indicate later model releases; purple are intermediate. Green lines represent uncertainty in true peak date/magnitude and black line is the seven-day rolling average. The convergence plot for peak date (subfigs B, E, and H) demonstrates the uncertainty in true peak date (green shaded region; median provided for reference) and the model's predictions of the peak date for each model release leading up to the peak. The convergence plot for peak magnitude (subfigs C, F, and I) demonstrates the range of true peak magnitude and the model's prediction of the peak magnitude for each model release leading up to the peak. The blue diamond represents first release the prediction was inside the green window (`PeakDate_FirstAccurate`) and the orange diamond represents the model is consistently inside the window of peak date/magnitude from that release onwards (`PeakDate_FirstConsistent`).

3 All model releases and recovery error plots

Section 3 of the Supplement presents all predictions from every model release, for all peak events. Additionally included are recovery error plots for peak events for which recovery occurred.

3.1 IHME daily deaths predictions

All predictions from every model release of the IHME model, for all daily deaths peak events are presented. Included are recovery error plots for peak events for which recovery occurred.

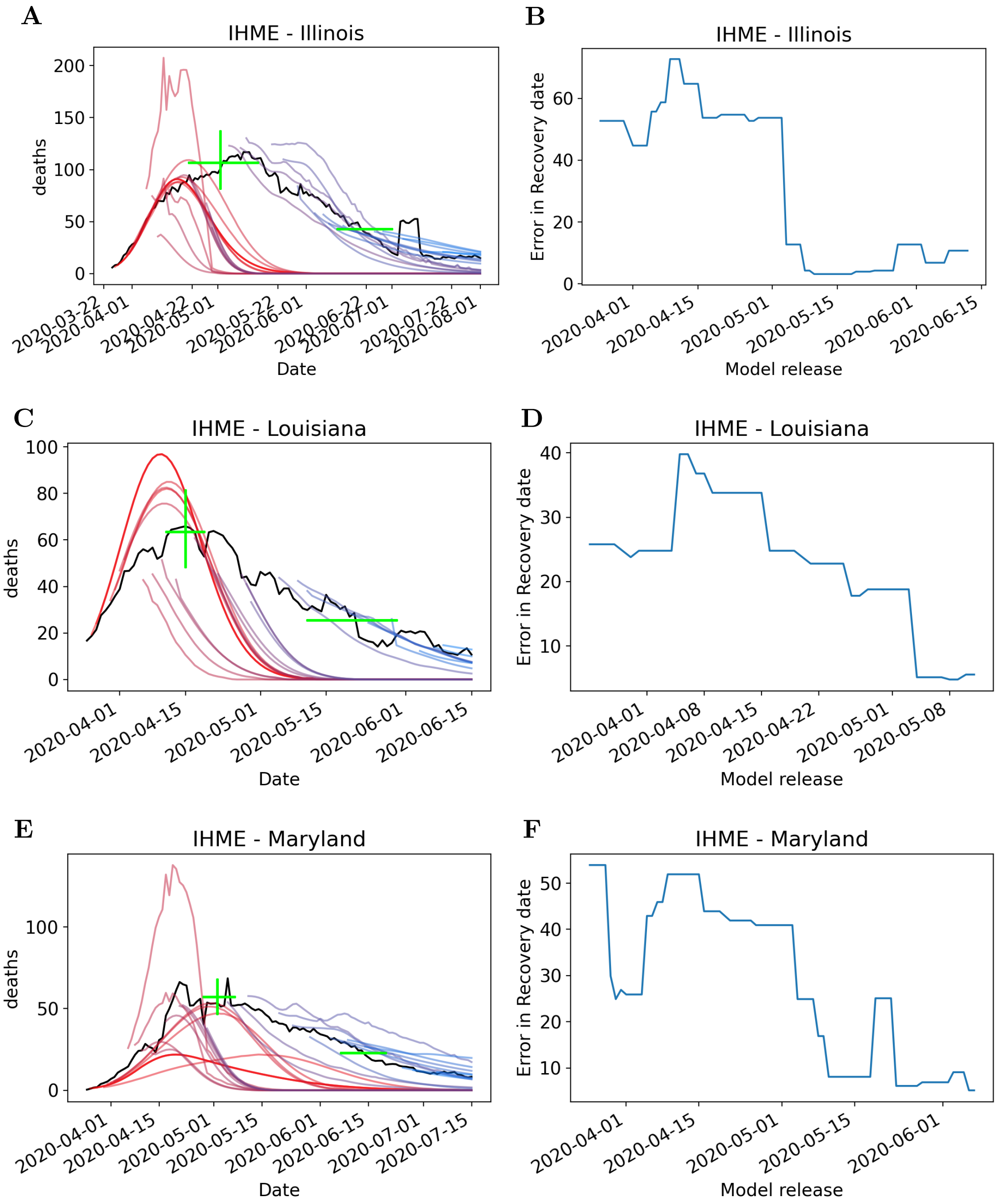

Figure E: Visualization of recovery predictions for Illinois, Louisiana, and Maryland during Spring 2020 of the IHME model. The IHME multi-release predictions are shown (subfigs A, C, and E) along with its error in prediction of recovery date (subfigs B, D, and F).

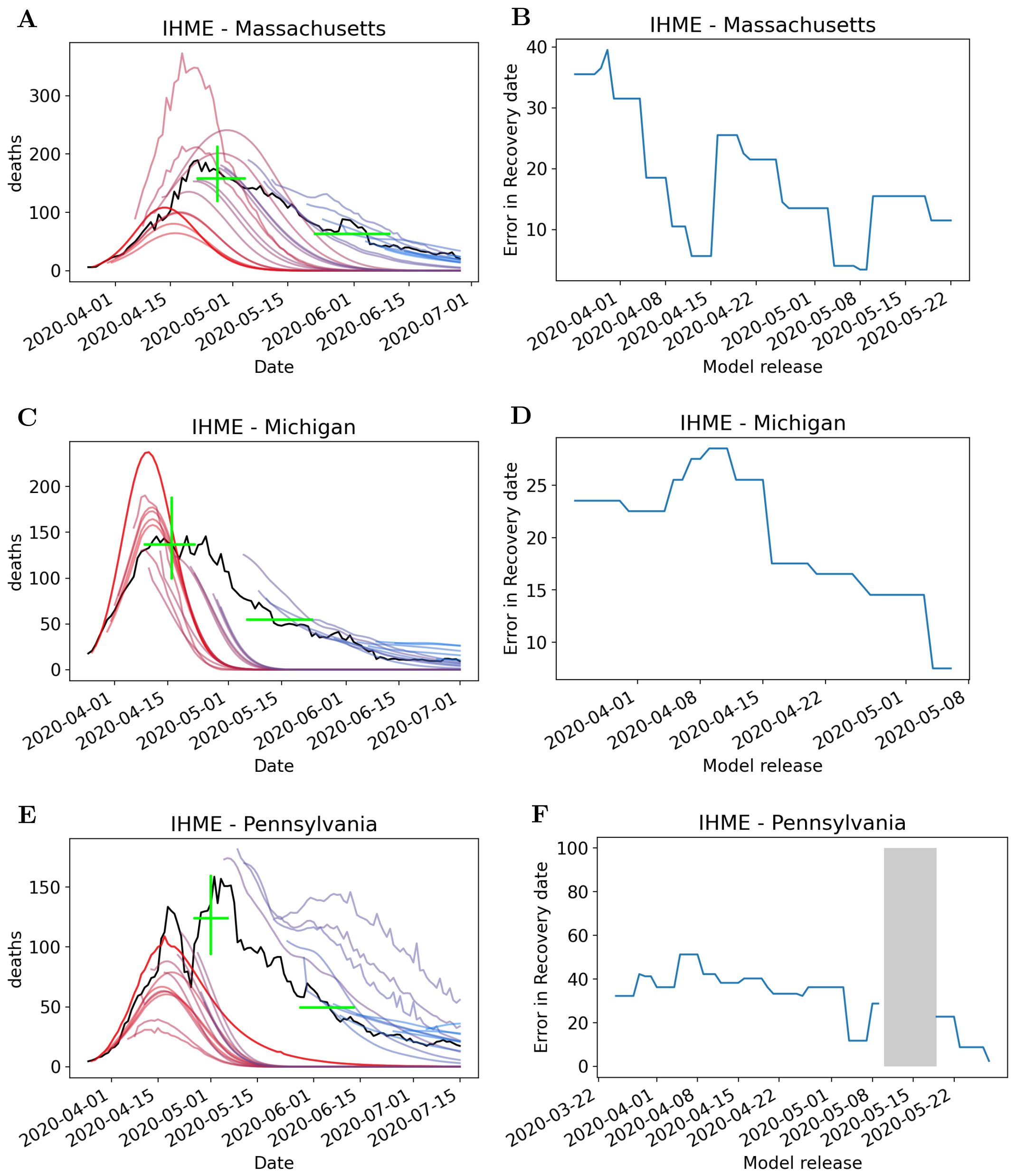

Figure F: Visualization of recovery predictions for Massachusetts, Michigan, and Pennsylvania during Spring 2020 of the IHME model. Shaded grey region indicates no date of recovery was predicted. The IHME multi-release predictions are shown (subfigs A, C, and E) along with its error in prediction of recovery date (subfigs B, D, and F).

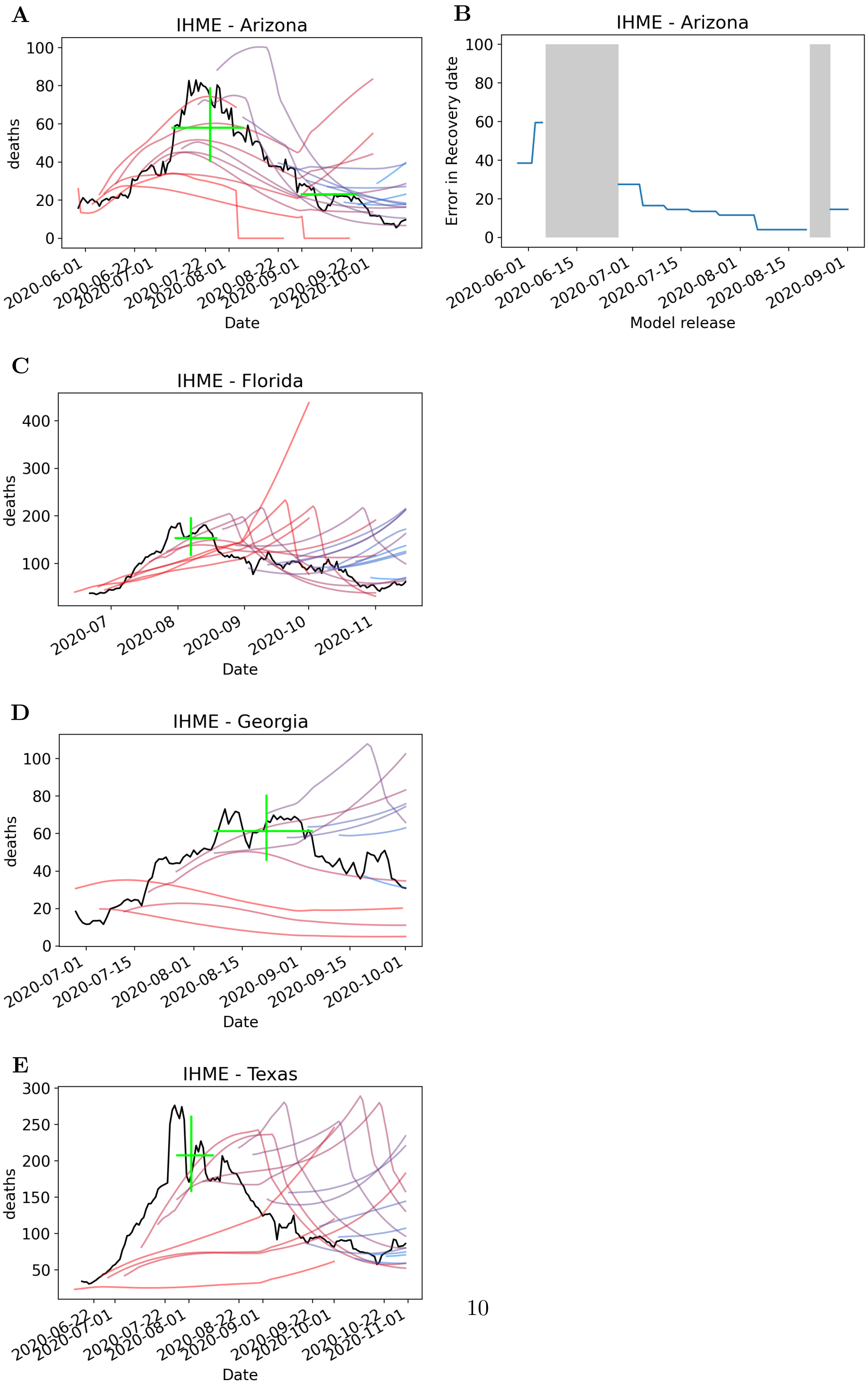

Figure G: Visualization of recovery predictions for Arizona, Florida, Georgia, and Texas during Summer 2020 of the IHME model. Shaded grey regions indicate no date of recovery was predicted. For the Arizona Summer 2020 peak IHME's multi-release predictions (subfig A) are shown along with its error in prediction of recovery date (subfig B). For the Florida Summer 2020, Georgia Summer 2020, and Texas Summer 2020 peaks no recovery occurred (subfigs C, D, and E).

3.2 Los Alamos daily deaths predictions

All predictions from every model release of the Los Alamos model, for all daily deaths peak events are presented. Included are recovery error plots for peak events for which recovery occurred.

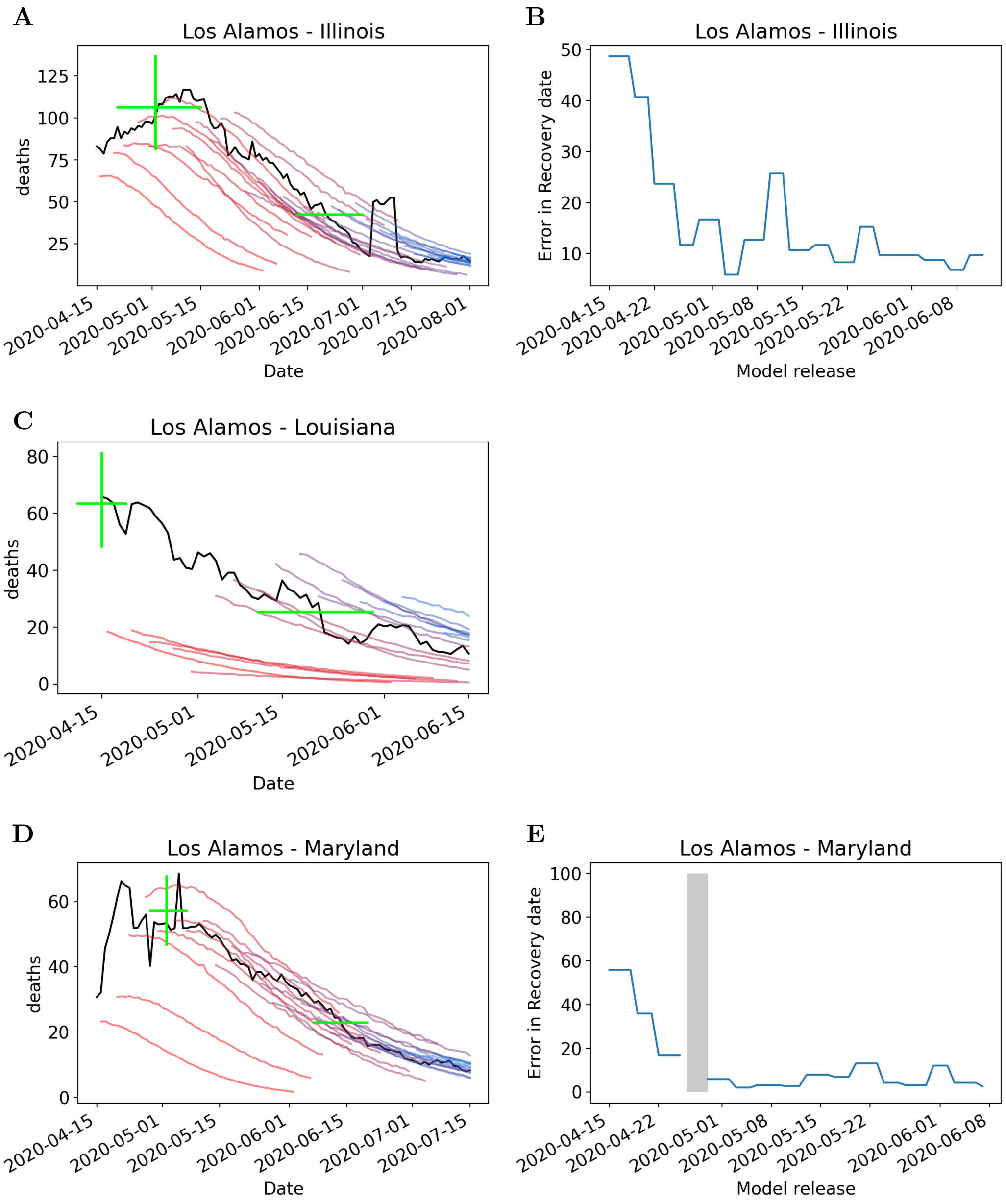

Figure H: Visualization of recovery predictions for Illinois, Louisiana, and Maryland during Spring 2020 of the Los Alamos model. Shaded grey region indicates no date of recovery was predicted. For the Illinois Spring 2020 and Maryland Spring 2020 peaks Los Alamos' multi-release predictions (subfigs A and D) are shown along with its error in prediction of recovery date (subfigs B and E). For the Louisiana Spring 2020 peak no recovery was predicted (subfig C).

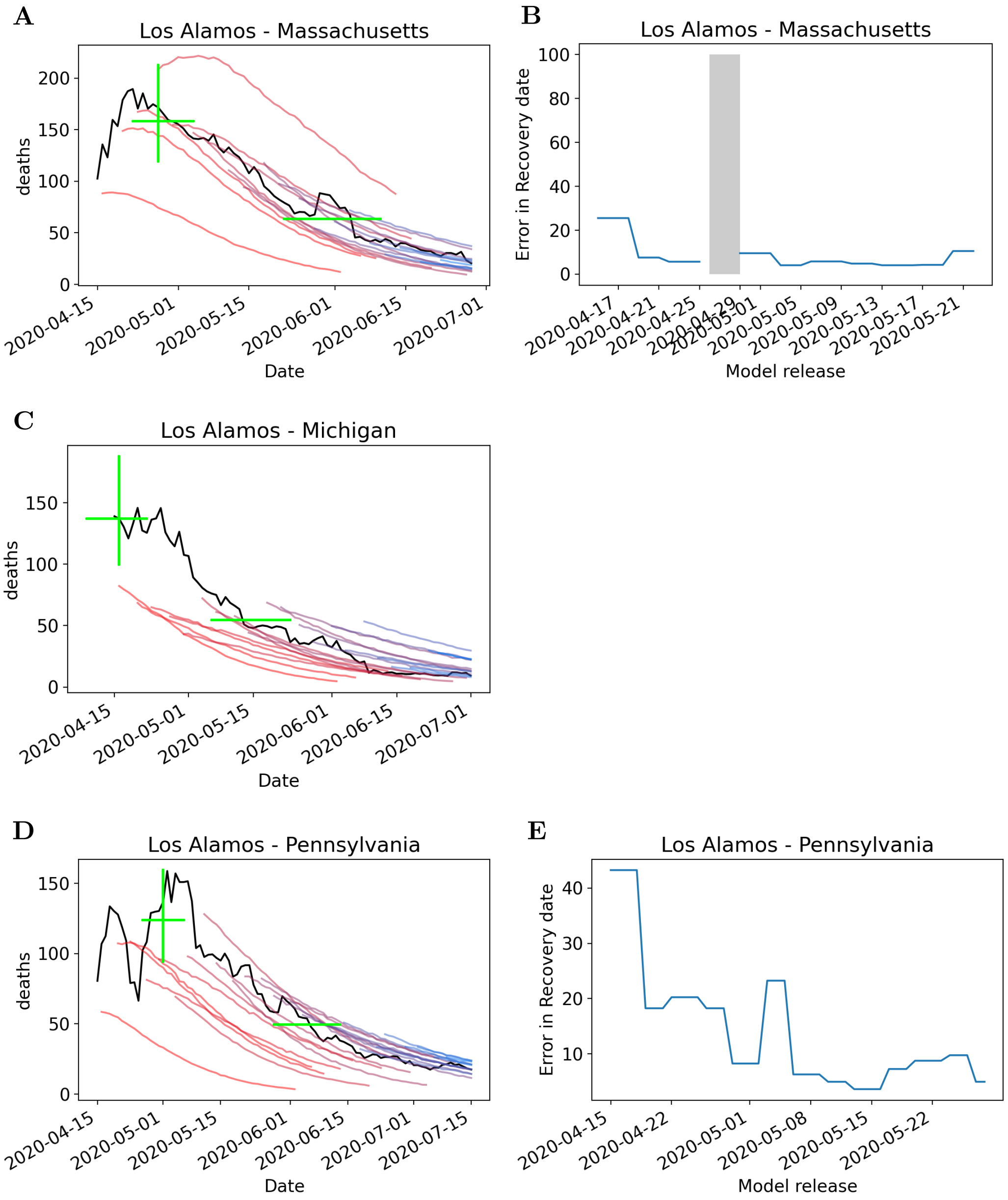

Figure I: Visualization of recovery predictions for Massachusetts, Michigan, and Pennsylvania during Spring 2020 of the Los Alamos model. Shaded grey region indicates no date of recovery was predicted. For the Massachusetts Spring 2020 and Pennsylvania Spring 2020 peaks Los Alamos' multi-release predictions (subfigs A and D) are shown along with its error in prediction of recovery date (subfigs B and E). For the Michigan Spring 2020 peak no recovery was predicted (subfig C).

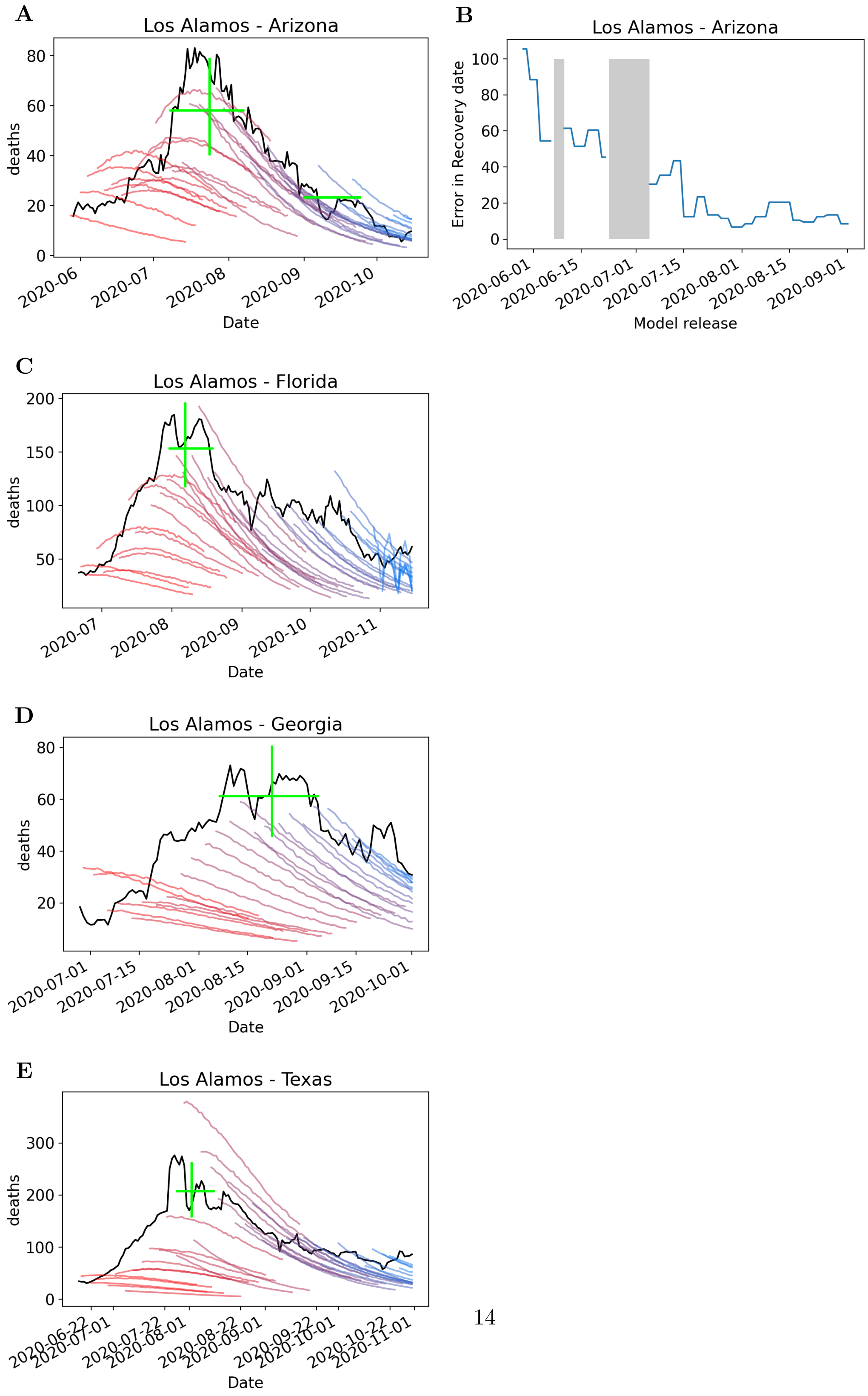

Figure J: Visualization of recovery predictions for Arizona, Florida, Georgia, and Texas during Summer 2020 of the Los Alamos model. Shaded grey regions indicate no date of recovery was predicted. For the Arizona Summer 2020 peak Los Alamos' multi-release predictions (subfig A) are shown along with its error in prediction of recovery date (subfig B). For the Florida Summer 2020, Georgia Summer 2020, and Texas Summer 2020 peaks no recovery occurred (subfigs C, D, and E).

3.3 UTexas daily deaths predictions

All predictions from every model release of the UTexas model, for all daily deaths peak events are presented. Included are recovery error plots for peak events for which recovery occurred.

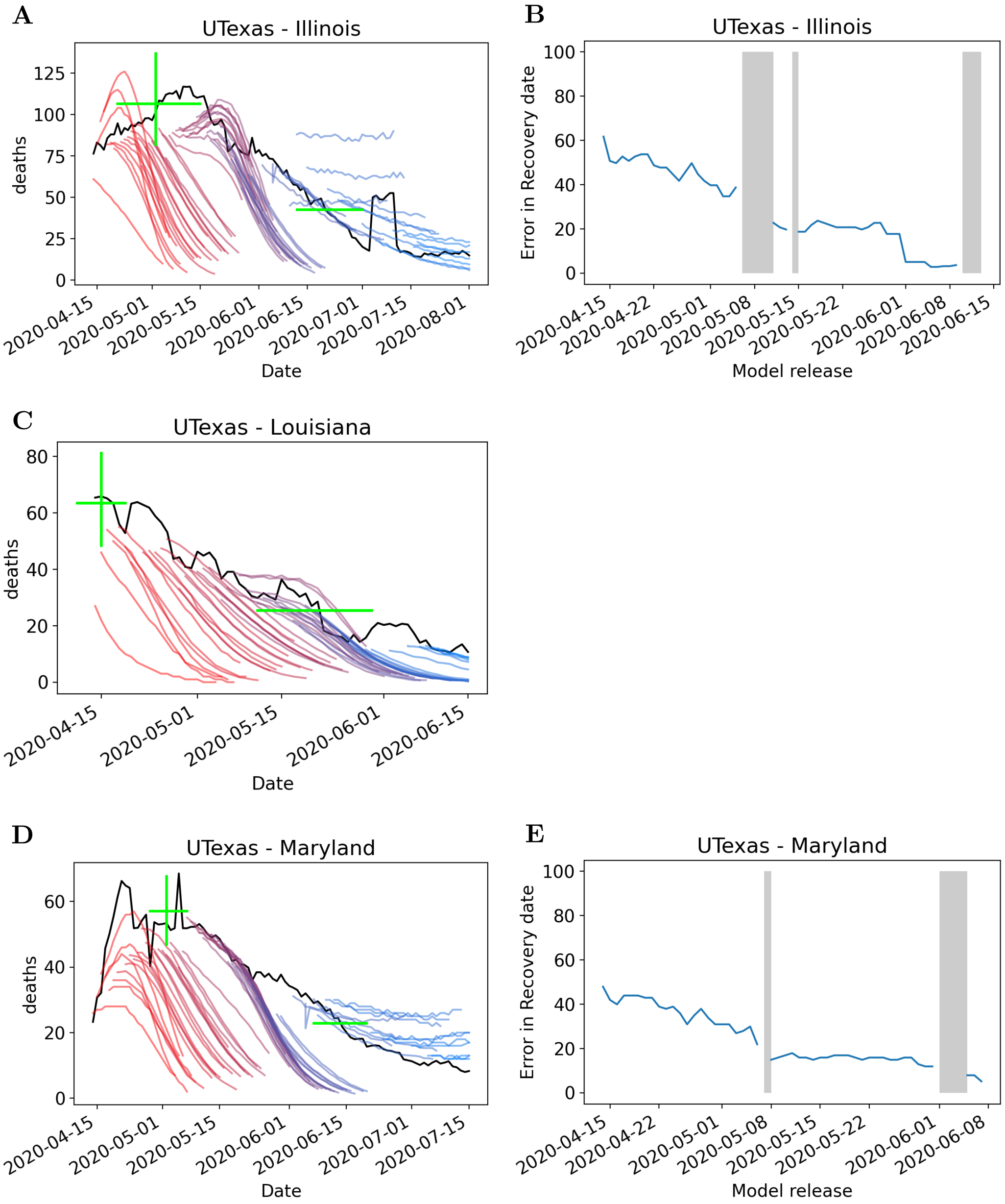

Figure K: Visualization of recovery predictions for Illinois, Louisiana, and Maryland during Spring 2020 of the UTexas model. Shaded grey regions indicate no date of recovery was predicted. For the Arizona Spring 2020 and Maryland Spring 2020 peaks UTexas' multi-release predictions (subfigs A and D) are shown along with its error in prediction of recovery date (subfigs B and E). For the Louisiana Spring 2020 peak no recovery was predicted (subfig C).

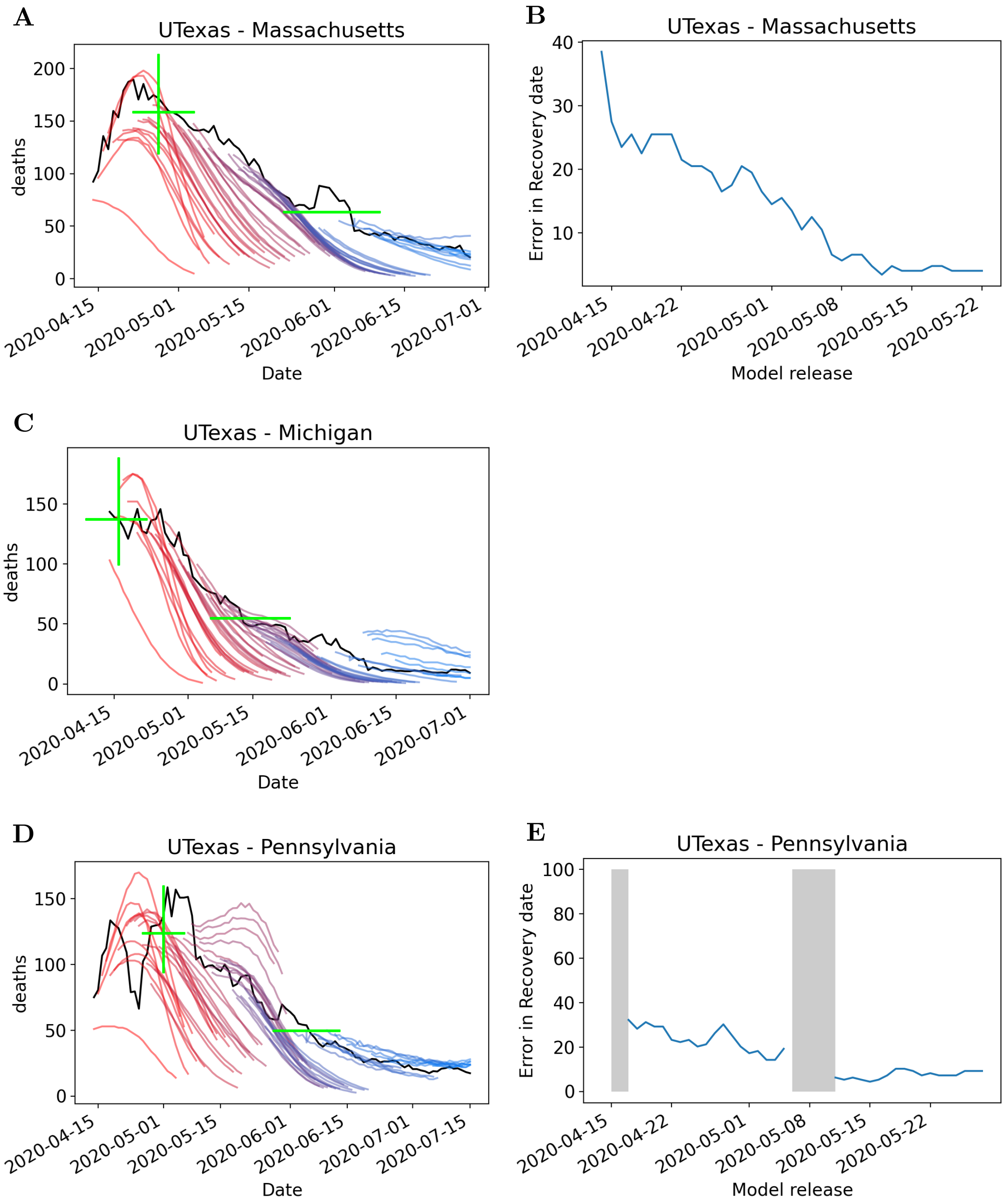

Figure L: Visualization of recovery predictions for Massachusetts, Michigan, and Pennsylvania during Spring 2020 of the UTexas model. Shaded grey regions indicate no date of recovery was predicted. For the Massachusetts Spring 2020 and Pennsylvania Spring 2020 peaks UTexas' multi-release predictions (subfigs A and D) are shown along with its error in prediction of recovery date (subfigs B and E). For the Michigan Spring 2020 peak no recovery was predicted (subfig C).

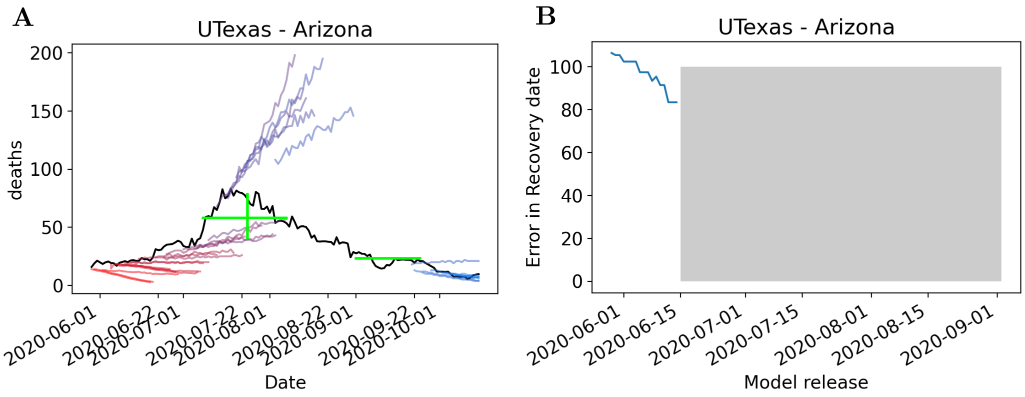

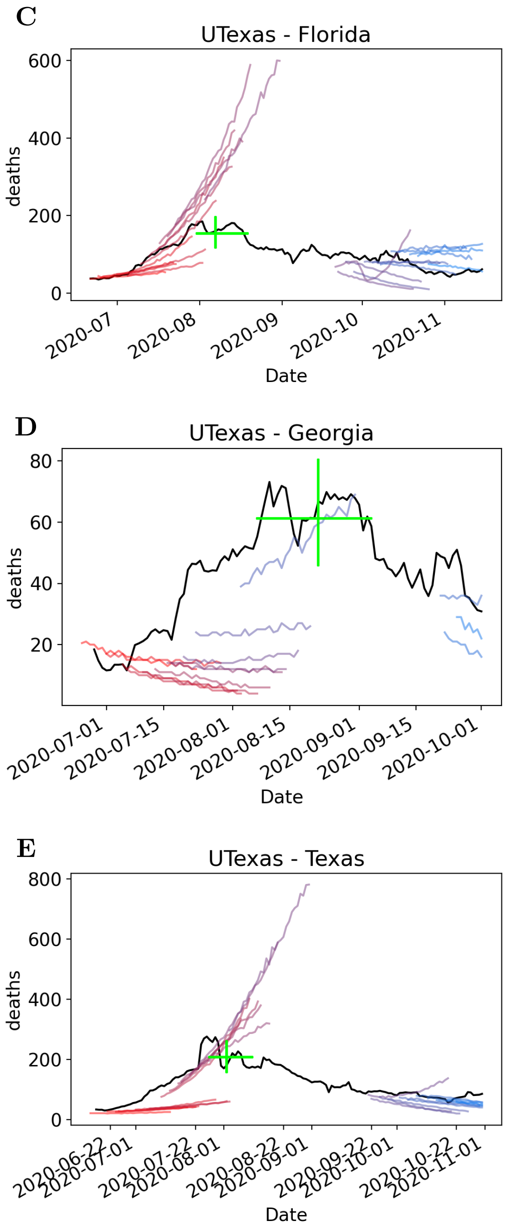

Figure M: Visualization of recovery predictions for Arizona, Florida, Georgia, and Texas during Summer 2020 of the UTexas model. Shaded grey region indicates no date of recovery was predicted. For the Arizona Summer 2020 peak Los Alamos' multi-release predictions (subfig A) are shown along with its error in prediction of recovery date (subfig B). For the Florida Summer 2020, Georgia Summer 2020, and Texas Summer 2020 peaks no recovery occurred (subfigs C, D, and E).

3.4 Youyang Gu daily deaths predictions

All predictions from every model release of the YYG model, for all daily deaths peak events are presented. Included are recovery error plots for peak events for which recovery occurred.

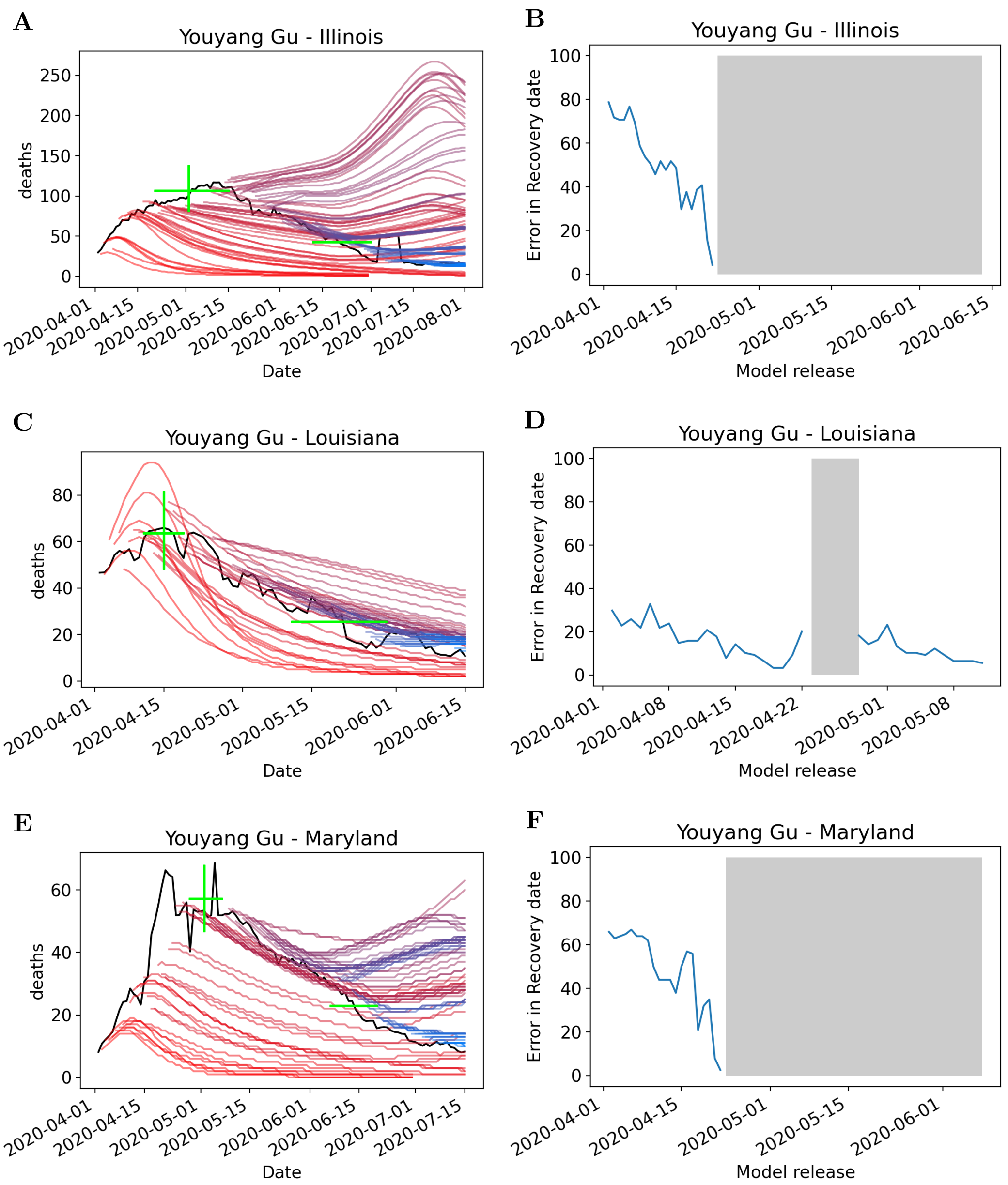

Figure N: Visualization of recovery predictions for Illinois, Louisiana, and Maryland during Spring 2020 of the YYG model. Shaded grey region indicates no date of recovery was predicted. The YYG multi-release predictions (subfigs A, C, and E) are shown along with its error in prediction of recovery date (subfigs B, D, and F)

.

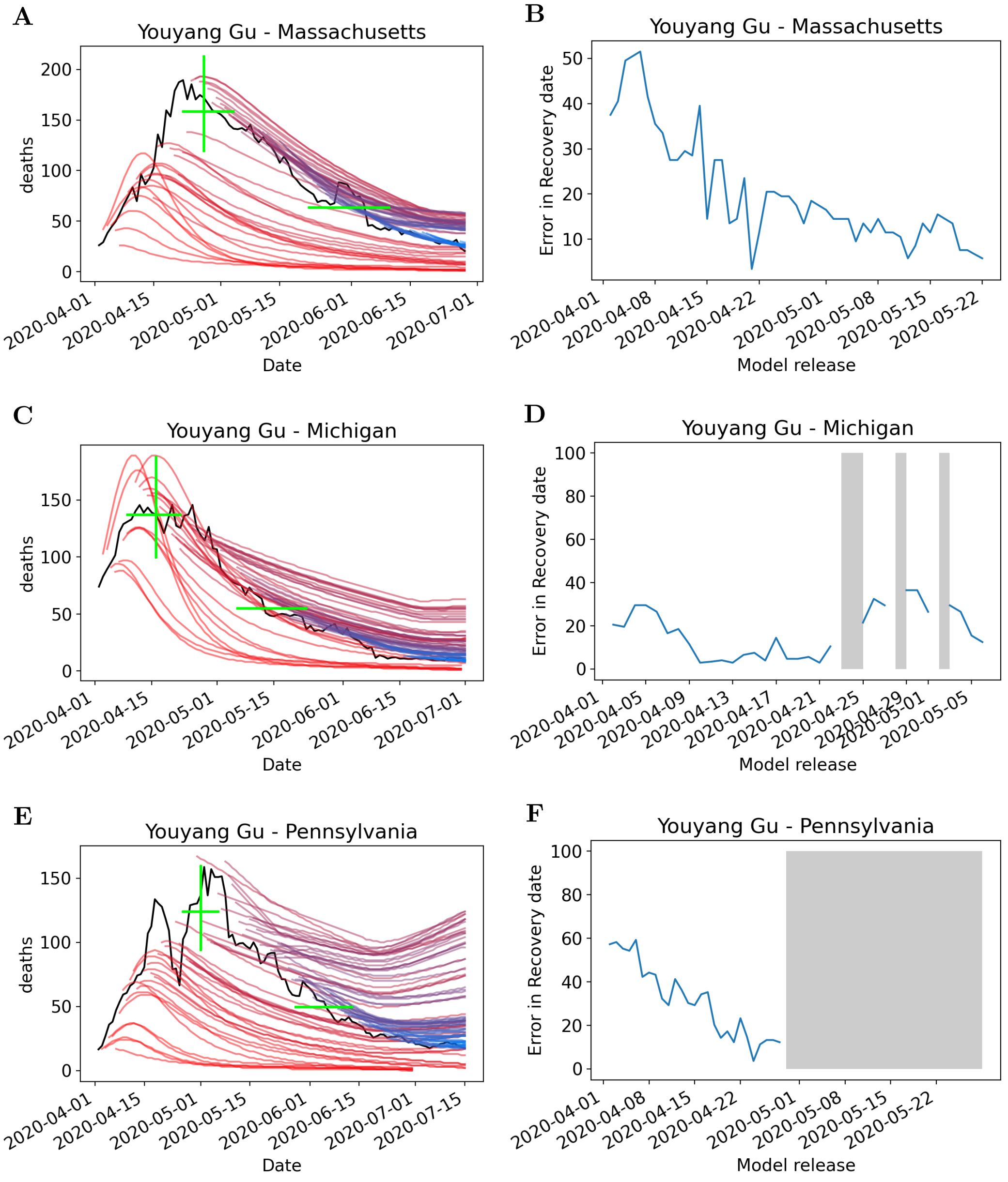

Figure O: Visualization of recovery predictions for Massachusetts, Michigan, and Pennsylvania during Spring 2020 of the YYG model. Shaded grey region(s) indicates no date of recovery was predicted. The YYG multi-release predictions (subfigs A, C, and E) are shown along with its error in prediction of recovery date (subfigs B, D, and F).

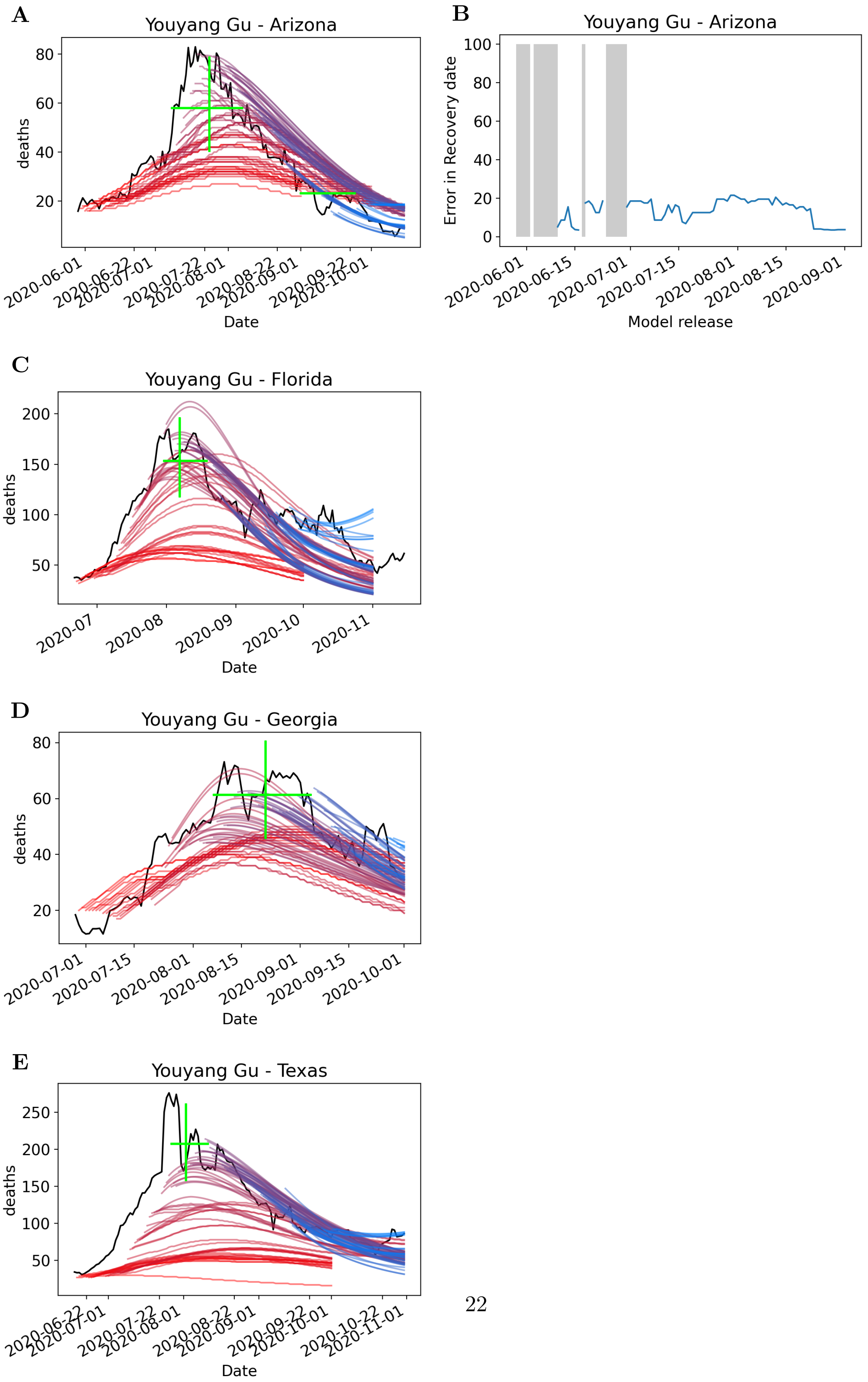

Figure P: Visualization of recovery predictions for Arizona, Florida, Georgia, and Texas during Summer 2020 of the YYG model. Shaded grey regions indicate no date of recovery was predicted. For the Arizona Summer 2020 peak YYG's multi-release predictions (subfig A) are shown along with its error in prediction of recovery date (subfig B). For the Florida Summer 2020, Georgia Summer 2020, and Texas Summer 2020 peaks no recovery occurred (subfigs C, D, and E).

3.5 IHME hospitalization predictions

All predictions from every model release of the IHME model, for all hospitalization peak events are presented. Included are recovery error plots for peak events for which recovery occurred.

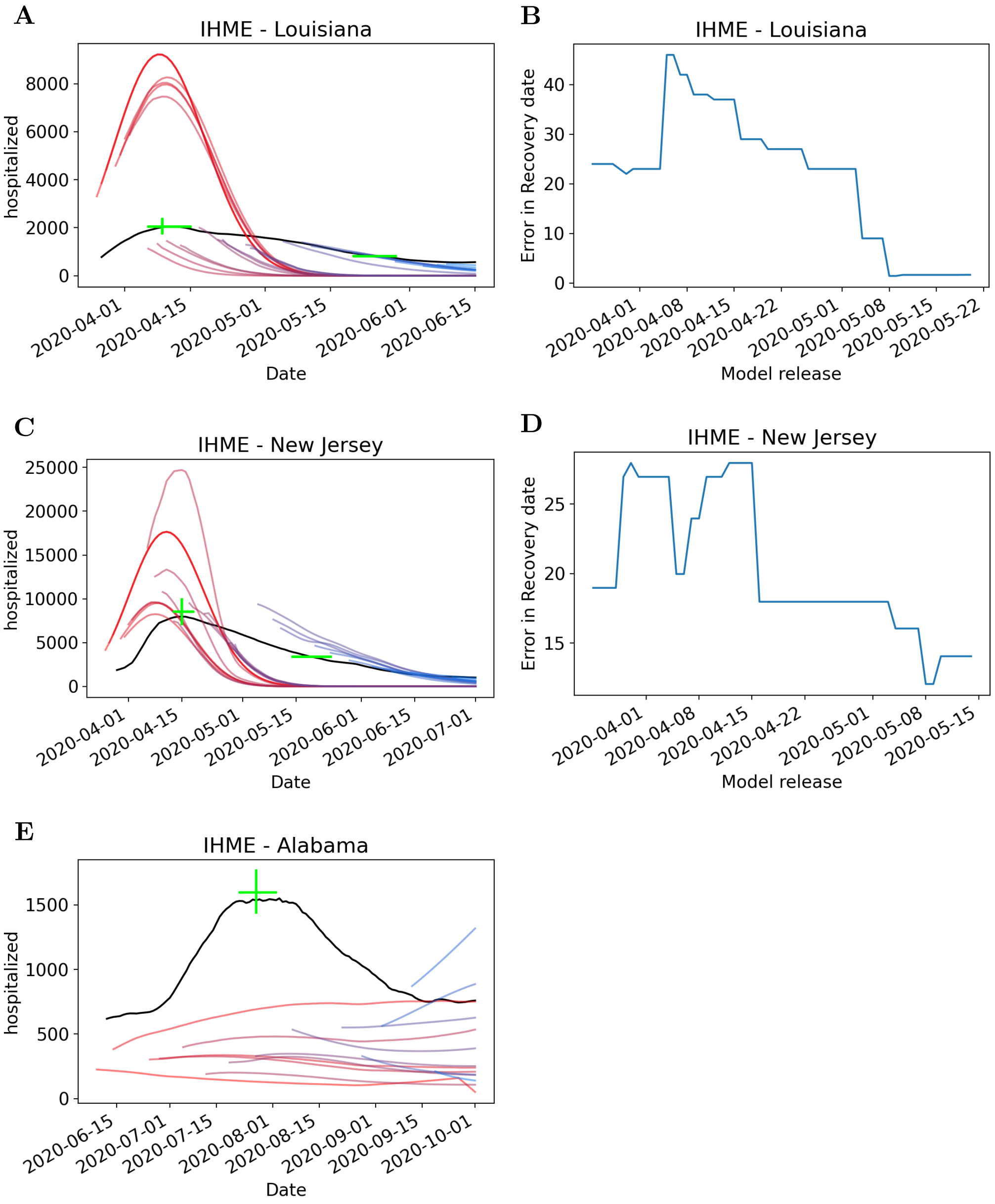

Figure Q: Visualization of recovery predictions for Louisiana Spring 2020, New Jersey Spring 2020, and Alabama Summer 2020 of the IHME model. For the Louisiana Spring 2020 and New Jersey Spring 2020 peaks IHME's multi-release predictions (subfigs A and C) are shown along with its error in prediction of recovery date (subfigs B and D). For the Alabama Summer 2020 peak no recovery occurred (subfig E).

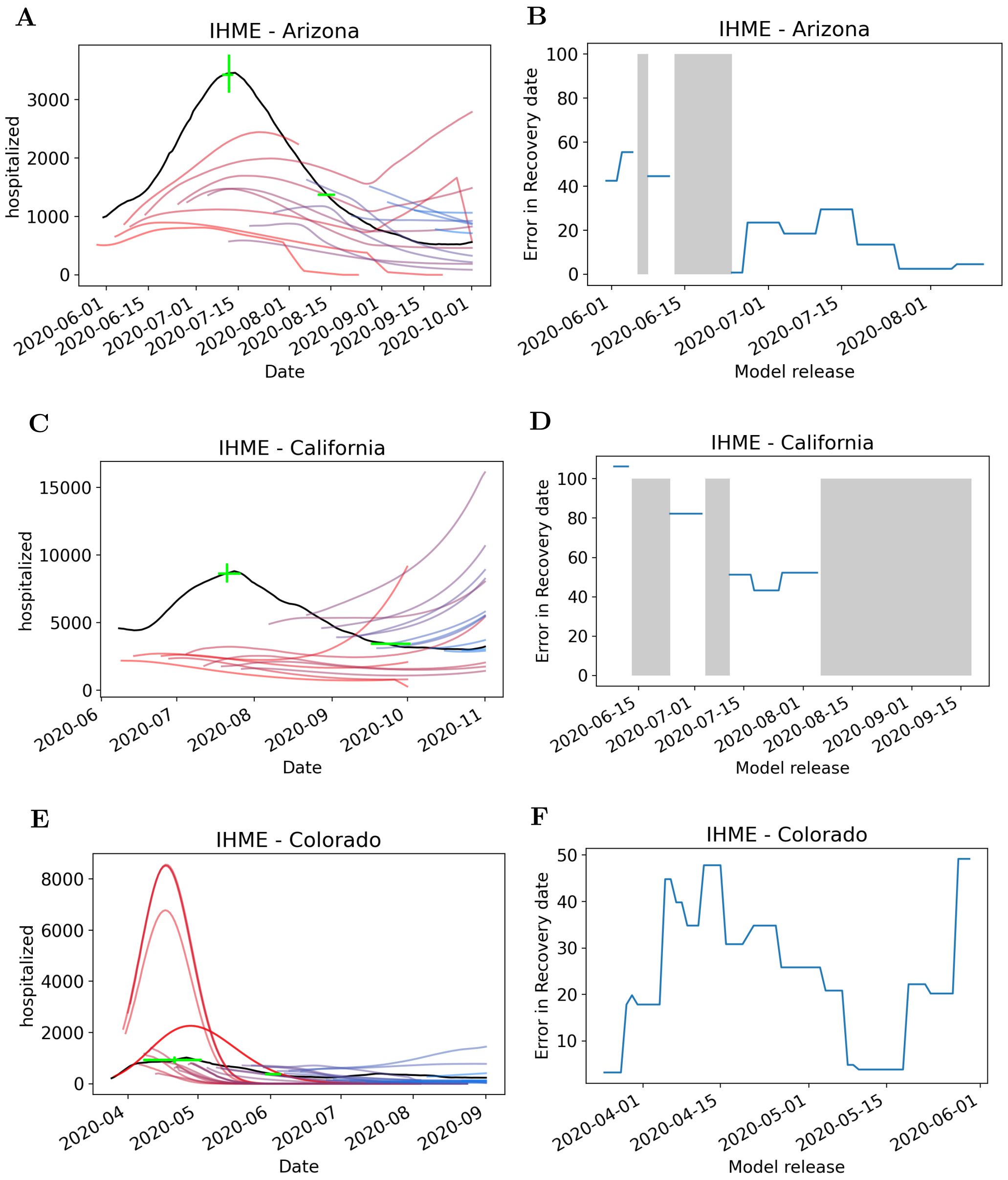

Figure R: Visualization of recovery predictions for Arizona, California, and Colorado during Summer 2020 of the IHME model. Shaded grey regions indicate no date of recovery was predicted. The IHME multi-release predictions (subfigs A, C, and E) are shown along with its error in prediction of recovery date (subfigs B, D, and F).

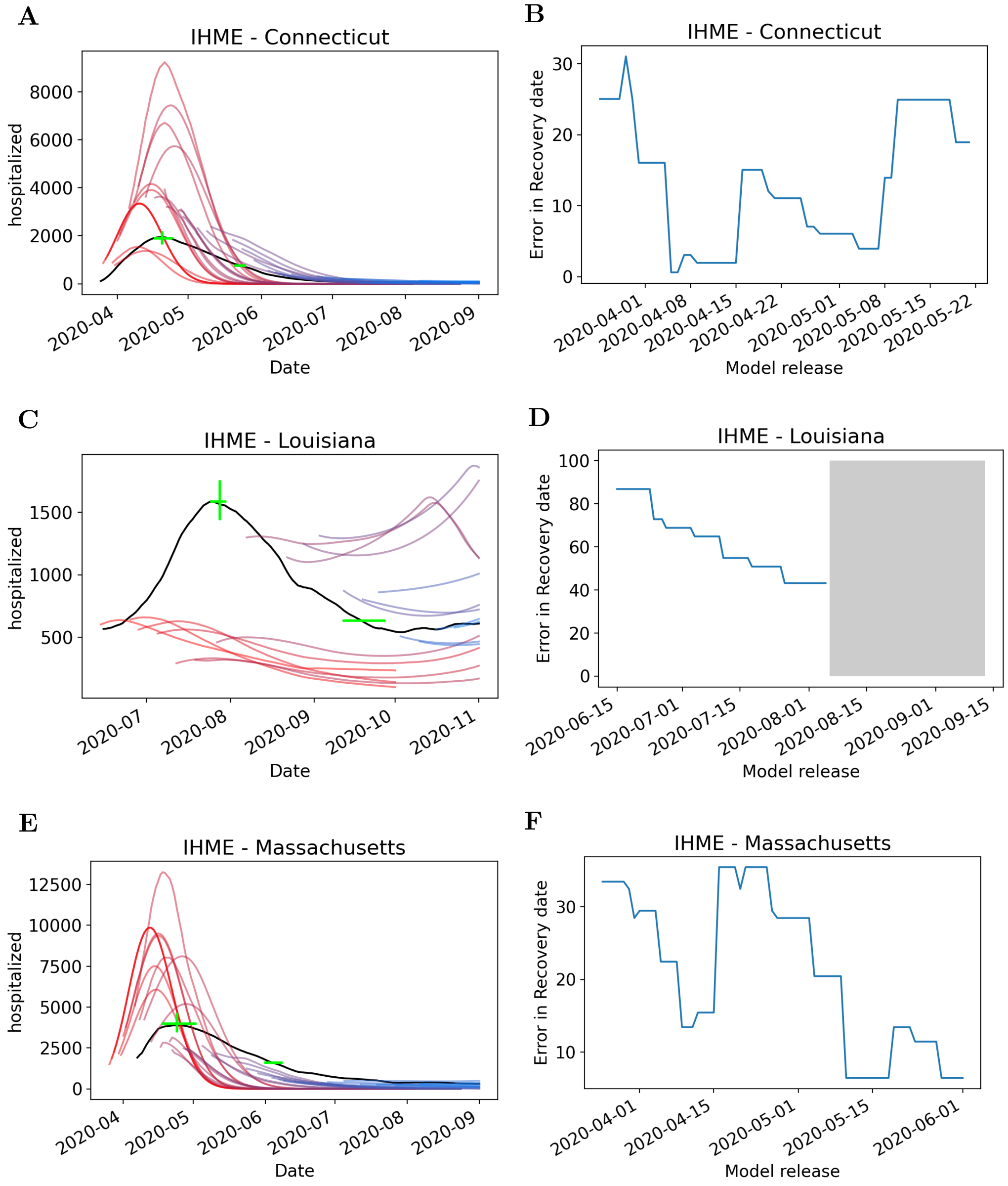

Figure S: Visualization of recovery predictions for Connecticut, Louisiana, and Massachusetts during Summer 2020 of the IHME model. Shaded grey region indicates no date of recovery was predicted. The IHME multi-release predictions (subfigs A, C, and E) are shown along with its error in prediction of recovery date (subfigs B, D, and F).

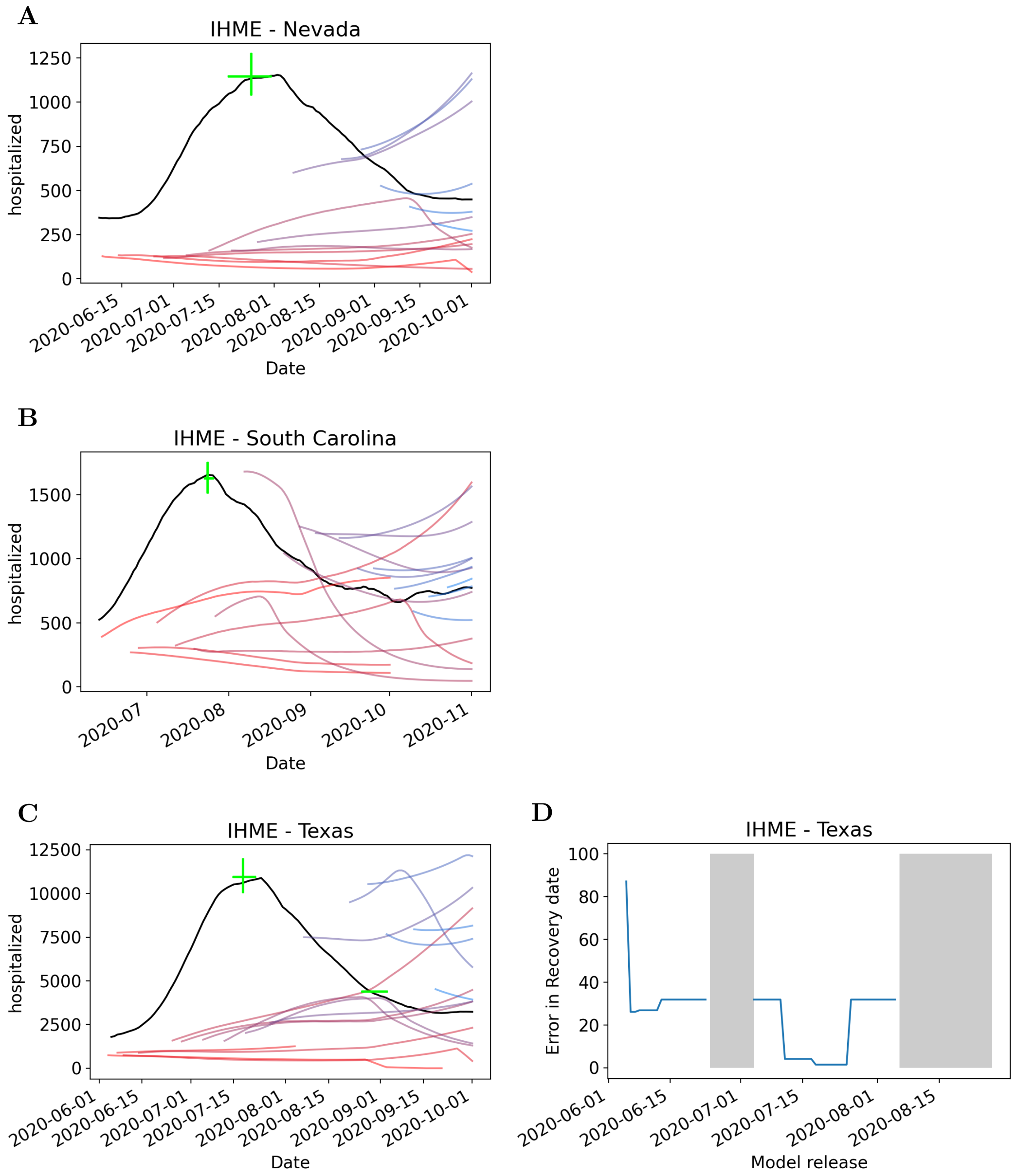

Figure T: Visualization of recovery predictions for Nevada, South Carolina, and Texas during Summer 2020 of the IHME model. Shaded grey regions indicate no date of recovery was predicted. For the Nevada Summer 2020 and South Carolina Summer 2020 peaks no recovery occurred (subfigs A and B). For the Texas Summer 2020 peak IHME's multi-release predictions (subfig C) are shown along with its error in prediction of recovery date (subfig D).
